# Norfloxacin Oxidative Degradation and Toxicity in Aqueous Media: Reciprocal Effects of Acidity Evolution on Metal Cations and Clay Catalyst Dispersion

**DOI:** 10.3390/ijms26094347

**Published:** 2025-05-02

**Authors:** Roumaissa Djidja, David Dewez, Abdelkrim Azzouz

**Affiliations:** 1 Nanoqam, Department of Chemistry, University of Quebec at Montreal, Montreal, QC H3C 3P8, Canada; djidja.roumaissa@courrier.uqam.ca; 2 Station Expérimentale des Procédés Pilotes en Environnement (STEPPE), École de Technologie Supérieure, Montreal, QC H3C 1K3, Canada

**Keywords:** metal cations, norfloxacin, montmorillonite, ozonation, *Lemna minor*, toxicity

## Abstract

The ozonation of norfloxacin (NOF), a widely used fluoroquinolone antibiotic, in the presence of Na^+^, Fe^2+^, Cu^2+^, Ni^2+^, and Co^2+^ cations and their montmorillonite-supported counterparts was investigated. The NOF degradation and the toxicity of the ozonized mixtures towards an aquatic organism (*Lemna minor*) were evaluated in terms of changes in its frond number, chlorophyll content, photosynthesis efficacy, and production of reactive oxygen species (ROS). The evolution over time of the NOF degradation grade and the toxicity were discussed in terms of *i.* the observed changes in the interactions of the cation and clay catalyst with NOF molecules; ***ii.*** the pH decay, during ozonation. Ion-exchange and Lewis acid–base interactions appear to govern NOF adsorption and clay catalyst dispersion in correlation with the progressive formation of acidic species in the aqueous media. These findings reveal promising prospects for tailoring optimum oxidative water treatments with minimum toxicity and for predicting their environmental impacts on aquatic media.

## 1. Introduction

Antibiotic overuse and release in nature, even at low concentrations, are now recognized as pollution sources with negative impacts on biodiversity, particularly in aquatic environments [1]. Antibiotics can not only accumulate in ecosystems, giving rise to antibiotic-resistant bacteria, but also undergo unavoidable decomposition into persistent derivatives that are harmful to aquatic life [2,3]. Fluoroquinolones (FQs) are the third-largest class of antibiotics and are commonly employed for both human and animal infection issues, and they have been ubiquitously detected in aquatic environments at various concentrations. In surface waters, ciprofloxacin and norfloxacin are frequently reported at levels ranging from ng/L to μg/L [4,5,6].

In aquatic ecosystems, plants and algae are vital to the biodiversity equilibrium, contributing through photosynthesis, oxygen production, nutrient cycling, and as food sources. They can also act as bioindicators for monitoring water quality due to their sensitivity and rapid response to pollutants. In plant metabolism, FQs were found to trigger the production of reactive oxygen species (ROS) that inhibit *C. ovalisporum* biomass growth and reduce the chlorophyll *a* content and photosynthetic capacity [7]. The resulting Chl triplet state can react with ^3^O_2_, yielding additional ROS that inhibit chlorophyll synthesis [8]. FQs’ quinolone ring structure gives them appreciable chemical resistance to hydrolysis and high thermal resistance [9]. Norfloxacin (NOF) is the most frequently detected FQ in wastewaters, groundwaters, and even in drinking water supplies [10]. This relative chemical and thermal stability mean that NOF is regarded as a potentially persistent organic pollutant that demonstrates bacteria resistance and has a prolonged impact on biodiversity. This is why particular interest was devoted to NOF oxidative degradation. NOF’s chemical structure (Scheme S1) includes an electron-rich piperazine moiety that displays reactivity, particularly towards ozone and other oxidizing agents [11].

Among the many methods for the oxidative removal of organic pollutants [9,12,13], ozonation in clay suspensions appears to be the most suitable method for achieving a nature-inspired oxidative technique that can mimic the catalytic activity of clay-rich media [14]. Clay-rich media govern not only the ionic force and the pH but also the complex [clay–substrate–water–cation] interactions, and subsequently the very kinetics, of the oxidative process [15]. For instance, the maximum adsorption of ciprofloxacin (CIP) onto montmorillonite K-10 and kaolin was observed at pH 7.4, presumably due to the strong electrostatic interactions between the zwitterionic form of CIP and the clay surface [16]. The maximum adsorption, involving π-π stacking interactions and H-bridges, between NOF zwitterions and polydopamine microspheres was also reported at the optimal pH 6.6 [17].

Montmorillonite (Mt) is a 2:1 clay mineral with aluminosilicate lamellae that can reciprocally interact and exhibit various interactions with species dispersed in aqueous media according to the pH level. Each lamella has two tetrahedral silica layers that sandwich an octahedral one, where Mg^2+^ substitutes for Al^3+^, generating a permanent negative charge balanced by exchangeable cations [18,19]. The lamella structure is also expected to bear both in-plane and out-of-plane silanols, which are located between the exchangeable cations and in the surface edges, respectively [20]. Both the silanols induce additional pH-dependent charges and hydrophilic characteristics, unlike silica-rich islands, which favor hydrophobic interactions with organic molecules through their siloxy groups. Moreover, the lattice oxygen atoms act as Lewis bases through their free electron pairs that promote Lewis acid–base interactions with metals cations and H-bridges with water and OH-functionalized molecules [21]. All these interactions are known to ***i.*** govern the clay sheet dispersion, interlayer water content, and exchangeable cation mobility, ***ii.*** modulate the accessible surface, ***iii.*** and favor adsorption and, subsequently, catalytic activity, as already reported for the clay-ozonation of organic pollutants [13,22,23,24,25].

Adsorption plays a crucial role in heterogeneous catalysis, particularly when metals are involved. Excepting alkalis and alkaline earths, along with iron in natural amounts, most metals are harmful for biodiversity beyond certain tolerated doses. Industrial and mining activities usually release cationic forms of heavy metals, such as Pb, Hg, Cd, Cr, Ni, and/or As, which are highly toxic to living organisms [26]. Other metals (Co, Fe, Cu, Mn, and Zn) are essential micronutrients for plants and animals at optimum concentrations but can become toxic beyond certain levels [27]. When released in wastewaters, metals can interact with organic pollutants, thereby influencing their degradation and toxicity evolutions. For instance, the Fe^2+^ cation is known to act as a catalyst in the oxidative degradation of organic molecules [28]. Furthermore, the addition of Fe_3_O_4_–MnO_2_ was found to enhance bisphenol A degradation, particularly in the presence of hydroxyl radicals (HO·) [29].

Nonetheless, the advanced degradation of organic molecules is often accompanied by toxicity changes due to the production of intermediates that could be more or less harmful than the parent molecules and that specifically interact with cations and adsorbent surfaces. In other words, changes in the product distribution unavoidably results in toxicity evolution during the degradation process in clay-containing media. In addition, metal cations unavoidably interact with the π electron-rich groups (carboxylic, carbonyl, and/or piperazinyl groups) of the molecules, thereby modifying their chemical properties and reciprocally reducing their bioavailability to living species and thus their respective toxicity [30,31,32,33].

The use of Fe^2+^, Zn^2+^, Cu^2+^, Ce^3+^, and Co^2+^ cations in oxidative degradation techniques must take into account the potential risk of metal leaching and of secondary contamination of the environment [34]. The Cu^2+^ cation was already found to inhibit the Na^+^/K^+^-ATPase enzyme in fish metabolism, resulting in reduced intracellular sodium absorption, cardiovascular collapse, and respiratory deficiency [35,36]. Reportedly, high thallium (Tl^+^) concentrations induce an increase in the oxidative stress level and the alteration of the photosynthesis activity of algal *Microcystis aeruginosa* [37]. Hexavalent chromium at certain pHs showed extreme toxicity that increased the oxidative stress on the plant *L. minor*, reducing the contents of sugar, chlorophylls, and carotenoids [38]. Exposure to the Co^2+^ cation induced oxidative damage in *L. minor,* with a deactivation of superoxide dismutase, SOD [39]. The presence of Zn^2+^ in plants may lead to a substitution of the central Mg^2+^ ion in chlorophyll, thus altering their photosynthetic core and system [40]. In *L. minor,* exposure to Ag^+^ affects the growth rate and the protection system that controls oxidative stress [41]. The toxicity of CuO is mainly due to the Cu^2+^ cation release in the media making contact with the plant roots [42,43]. Nickel appears to inhibit the photosystem of and induce oxidative stress in the microalgae *Phaeodactylum tricornutum* [44]. However, current toxicity risk assessments often overlook mixture interactions, underestimating the true ecological impacts. A comprehensive understanding of the combined toxicity effect of metals with NOF derivatives from oxidative degradation on aquatic organisms is useful in this regard.

This research targets knowledge advancement that provides foundations for designing practical applications involving nature-inspired and clay-catalyzed oxidative water treatments. This is useful for a better understanding of the role of clay-containing media in nature self-regeneration. A previous study demonstrated the proof of concept that clay minerals exhibit specific catalytic activity and selectivity in the oxidative degradation of antibiotics, and that the distribution of the products governs the toxicity evolution. The aim of the present study resides in correlating the role of metal cations to the catalytic activity and selectivity in NOF oxidative degradation by ozonation and demonstrating that the process evolution modifies the dispersion, activity, and selectivity of the clay catalyst behavior in aqueous suspensions and, consequently, the toxicity of the resulting mixture on aquatic ecosystems. For this purpose, *Lemna minor*, a macrophyte with rapid growth and easy cultivation [45], was used as a reliable bioindicator of environmental pollution [14,46]. Different criteria, such as the frond number (FN), chlorophyll content, and ratio, along with the ROS level, should provide valuable data regarding the decay in plant growth and photosynthetic efficiency [47,48,49,50].

## 2. Results and Discussion

### 2.1. Effect of Adjusted Initial pH on Non-Catalytic Ozonation

**Figure 1:**
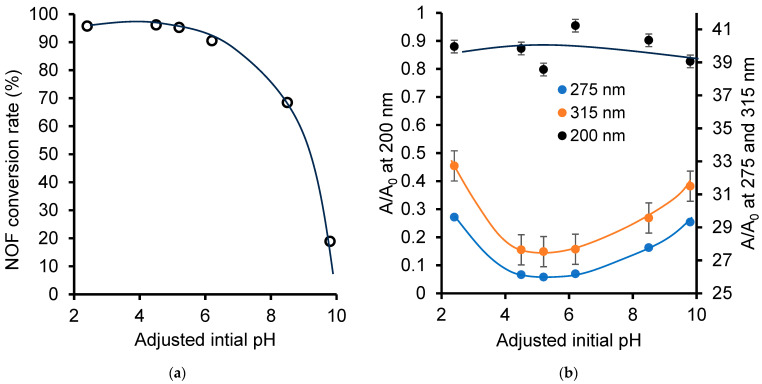
Effect of adjusted initial pH on relative UV–Vis band of NOF (**a**) and NOF conversion rate, as assessed through HPLC-DAD after 10 min of non-catalytic ozonation (**b**). The adjusted initial pH was measured with a ±0.2 accuracy that is overlapped by the mark width.

This research was first focused on the influence of the medium pH, regarded as a key-factor in the ozonation process, before investigating its effect on metal cations which already govern the clay catalyst structure (Figure S1). It clearly appears that pH changes strongly influence the process evolution and selectivity, most likely due to changes in the ozonation action mechanisms from molecular to radical forms, and vice versa [23,51]. Deeper insights in this regard through additional non-catalytic attempts during the 10 min ozonation at the adjusted initial pH showed a beneficial effect of the acidic pH on the oxidative degradation process (Figure 1a).

Indeed, lowering the initial pH from 9.8 down to 5.2–6.2 resulted in a pronounced improvement of NOF conversion from 18.92% up to a maximum plateau at 95.25–96.19%, with no changes below pH 5.2 down to 2.4. A possible explanation is that the non-catalytic ozonation of NOF is more favored by the acidic initial pH, which promotes the action of molecular ozone, than by neutral-to-basic media that favor hydroxyl radical-based pathways [23,51,52]. The pH changes within this range turned out to greatly influence the process selectivity, as illustrated by the evolution of the main UV–Vis bands of the ozonized NOF mixture (Figure 1b).

The pH changes seem to weakly influence the formation of OH-bearing species, including acids, and at most their interactions in water, given the barely detectable bump on the 200 nm band intensity between pH 4 and 6. By comparison, the pronounced minima of the 275 and 315 nm bands noticed in the same pH range must arise from the weak alteration of the other moieties of NOF molecules, including ring cleavage. This is probably due to a transitional pH range between the decreasing action of molecular ozone on the one hand and the increasing hydroxyl radical contribution on the other hand, and vice versa [23,51,52]. Such transitions induced by pH changes unavoidably control the contributions of the different potential reactions involved in the global ozonation process.

The non-catalytic ozonation of NOF induced a significant intensity increase for the 200 nm UV–Vis band (Figure S2a) and a decay for those appearing in the 250–370 region after only 10 min (Figure S2b). The pH decrease during ozonation is a precise indicator of the process advancement, providing evidence of the conversion of the organic substrates and the formation of acidic species but not necessarily of the process selectivity [53]. This is a common feature of all oxidative degradation reactions of organic molecules, regardless of the absence or presence of catalysts, the latter being mainly known to govern the process kinetics and selectivity. Therefore, one must expect that for a given imposed initial pH, the unavoidable decrease in pH during ozonation will influence the evolution of the process selectivity according to the presence/absence of catalysts, the process progress in time, and the intermediate distribution. This will be examined further.

### 2.2. pH Changes During Cation-Catalyzed Ozonation

Ozone induced a pH decrease in both pure water and the NOF solutions, which was even more pronounced in the presence of dissolved cations (Table 1). In pure water, this pH decrease must arise mainly from a two-step reaction of molecular ozone with hydroxyl radicals (O_3_ + HO^•^ → O_2_ + HO_2_^•^ ↔ O_2_^•^ − + H^+^) produced by a direct ozone reaction with water (O_3_ + H_2_O → 2HO^•^ + O_2_) [54]. In the absence of chemical species, including organic impurities, metal cations, carbonates and others, that consume ozone, pure deionized water is known to produce the most pronounced pH decrease during ozonation. Indeed, nanopure water ozonation gave a stronger pH decrease, which is expressed as a much higher pH difference (2.9) as compared to those registered for cation solutions, which range from 0.66 for Fe^2+^ to 1.39 for Na^+^.

The ozonation of NOF-containing media generates acidic species that are responsible of the pH decrease, which appears to be enhanced by the conversion rate of the organic substrate in the presence of dissolved cations (Figure S3a) and dispersed clay catalyst suspension (Figure S3b). As expected, this process appears to be enhanced by the catalytic role of the dissolved cation, as already reported for other organic substrates [22,24,25,28,53,55,56,57]. Cations act as Lewis acids, and once dissolved in water, they unavoidably undergo hydrolysis, resulting in an acidic solution. Hydrated cations will then act as Bronsted acids, releasing more or less hydronium ions (H_3_O^+^) according to their polarizing power (PP) [58]. That is why the initial pH of the cation solutions increased almost linearly (Ni^2+^ < Cu^2+^ < Co^2+^ < Fe^2+^ < Na^+^), with decreasing PP in the sequence Ni^2+^ > Cu^2+^ > Co^2+^ > Fe^2+^ >> Na^+^. A slight pH increase occurred after NOF addition, but the sequence was maintained. This is due to partial cation capture via two mechanisms: *i.* chelation by NOF atoms bearing accessible electron pairs; *ii.* cation exchange, given the presence of negative charges on NOF molecules at these pH levels.

**Table 1:** Initial features of cation-containing NOF solution, with pH values from before and after 10 min ozonation.

Metal Cation	Dissolved Cation ^a^		Aqueous Medium	Initial Charges of NOF ^c^		pH		
	PP ^a^	Lewis Acidity ^b^						
				R_2_N	-CO_2_^−^	Initial ^d^	Final ^e^	Diff. ^f^
None	X	X	Water	X	X	6.50	3.60	2.9
			NOF_(aq)_ ^c^	+++	-	5.28	2.41	2.87
Na^+^	1.0	0.16	Water	X	X	7.49	6.10	1.39
			NOF_(aq)_^c^	++	--	6.13	4.67	1.46
Fe^2+^	3.3	0.34	Water	X	X	6.16	5.50	0.66
			NOF_(aq)_^c^	+	---	6.8	3.15	3.65
Co^2+^	3.6	0.40	Water	X	X	6.04	4.88	1.16
			NOF_(aq)_^c^	++	---	6.29	3.14	3.15
Ni^2+^	4.2	0.50	Water	X	X	5.24	4.50	0.74
			NOF_(aq)_^c^	+++	--	5.81	3.09	2.72
Cu^2+^	3.8	0.45	Water	X	X	5.83	4.52	1.31
			NOF_(aq)_^c^	++	---	6.26	3.18	3.08

^a^ PP: polarizing power of cations, as expressed in (Charge)/(Ionic radius in Å)^2^; ^b^ Lewis acidity strength as expressed in valence units (v.u.), as defined in [58,59]; ^c^ NOF_(aq)*_: solution concentration: 3.31 × 10^−6^ M; ^c^ arbitrary charge scale based on pKa values of 8.75 and 6.34 for ammonium and carboxyl groups of NOF, respectively: 1. +++ or ---: >50%, 2. ++ or --: ~50%, 3. + or -: <50%; ^d^ initial pH before ozonation; ^e^ final pH after 10 min ozonation. All the pH values were measured with an average standard deviation of 0.01; ^f^ pH difference between the initial and final values during ozonation. X: feature not applicable.

**Figure 2:**
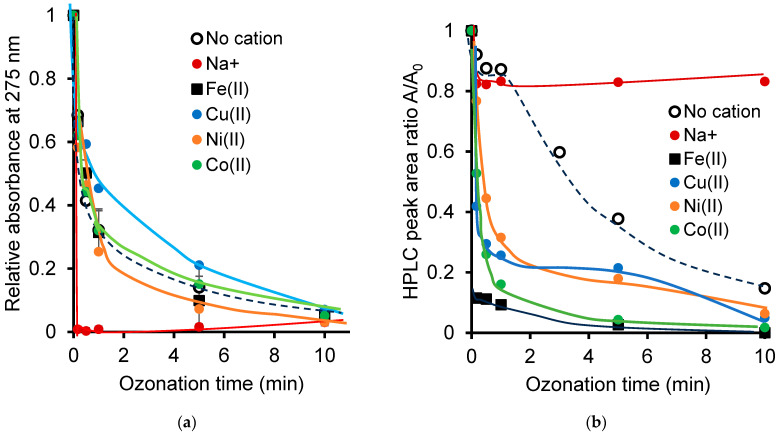
The time evolution of the main UV–Vis band of NOF (**a**) and the NOF conversion yield as determined by HPLC-V during cation-catalyzed ozonation (**b**). The adjusted initial pH was measured with a ±0.2 accuracy that is overlapped by the mark width. The HPLC-DAD peak area ratio was evaluated with a standard deviation of ±0.02. The dashed lines account for process evolutions during ozonation in cation-free media.

In NOF ozonation, this pH decay was found to decrease in the following sequence Fe^2+^(3.65) > Co^2+^(3.15) > Cu^2+^(3.08) > water (2.87) > Ni^2+^(2.72) > Na^+^(1.46) and is potentially due to the production of acidic ozonation product. Unlike Na^+^ cation, nanopure water seems to behave similarly to the other cations, as reflected by similar decays in the 275 nm band absorbance (Figure 2a) and residual HPLC peak ratio of NOF during ozonation (Figure 2b).

Here, Na^+^ cation seems to mitigate NOF oxidative degradation by ozone, as supported by the plateau of the residual NOF HPLC peak ratio after a weak depletion by less than 20% after a few seconds of ozonation. In the meantime, this specific behavior of the Na^+^ cation appears to favor selectivity towards certain NOF reactions, as reflected by the sharp decay and almost total disappearance of the relative absorbance of the 275 nm band of NOF. Besides, the Na^+^ cation is expected to promote an indirect ozonation of NOF through a higher hydroxyl radical production than any other transitional metal cations, particularly at the starting neutral-to-alkaline pH levels [60].

This is presumably due to a complete chelation process with NOF molecules, as reflected by the total disappearance of the UV–Vis spectrum of NOF in the NaCl solution (Figure S4). Nonetheless, this was accompanied by a sharp intensity increase and broadening for the 210–220 nm band of NOF, as with any other cation. Hence, it clearly appears that dissolved cations influence the course and selectivity of NOF ozonation, which allows the prediction of an influence on the distribution of the ozonation products and the toxicity for the targeted bioindicator. This will be investigated further.

### 2.3. pH Changes During Clay-Catalyzed Ozonation

**Figure 3:**
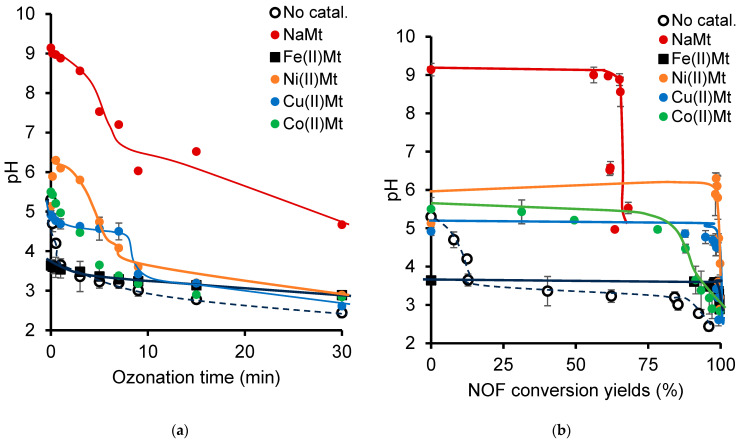
The evolution of instant pH (**a**) and correlation as a function of NOF consumption during ozonation (**b**). Ozone flow rate: 600 mg/h. Catalyst amount: 2 g/L. Sample volume: 25 mL. NOF concentration: 3.31 × 10^−6^ M. The NOF conversion yield was calculated using the relationship (1 − (A/A_0_)) × 100% and the measurement of the instant/initial peak area ratio (A/A_0_) by HPLC-DAD. The instant pH was measured with a variable standard error (±0.2–0.5) during ozonation.

As previously stated, significant changes in the UV–Vis spectrum were observed after only 10 min of NOF in water (Figure S2). Simultaneously, the initial pH decreased from 5.28 to approximately 3.65 after only 1–2 min (Figure 3a) for an NOF conversion not exceeding 10–12%, most likely due to an early formation of acids. A correlation attempt revealed that the pH of the ozonation mixture remains constant until certain threshold values of the NOF conversion yield (Figure 3b). Non-catalytic ozonation showed a long and slightly inclined plateau to a pH of 3.2 and an abrupt drop down to 2.44 for NOF conversions of ca. 84.12% and 95.96%, respectively. The shape of the pH profile suggests the occurrence of a two-step ozonation process with NOF conversion, mainly into acid species, sandwiching an intermediate step with less acid production.

The pH decline (Figure 3a) seems to be non-dependent on the NOF degradation increase, as illustrated by a specific plateau for each clay catalyst, which was slightly shorter for NaMt (Figure 3b). This must be due to the occurrence of buffering effects involving the progressively generated acidic species and their instant and unavoidable reaction with the metal cations of the clay catalysts into carboxylate salts. The continuous formation of oxidized species was supported by the intensity increase for the 200 nm UV–Vis band, which resulted from clay catalyst addition (Figure S5). Metal cation exchange between the clay catalysts and NOF molecules and then the generation of acidic species were demonstrated by the ICP-OES analysis via the changes in the cation amounts in the NOF solution before and after ozonation (Figure S6). The NOF molecules, and then most of its oxidized derivatives, bear Lewis’s base sites and carboxyl groups, which exert competitive chelation and the ion exchange of metal cations at the expense of clay catalysts, depending on the pH level. Both phenomena prevent the metal cation from being released into the bulk solution, thereby explaining why no significant iron loss was detected by the ICP-OES during ozonation, at least up to certain pH levels [61,62,63,64,65]. However, the excessive pH decrease during ozonation and the increasing number of re-use cycles promote irreversible cation loss. Unless altered upon acid attack, the clay structure can be revived through periodic impregnations in fresh metal cation solution [22].

In the meantime, a marked NOF depletion occurred during the first 2–7 min of ozonation, as illustrated by the pronounced intensity decrease of the HPLC-DAD peak ratio of NOF by more than ca. 90%, which was much weaker (ca. 63–65%) with NaMt or in the absence of catalyst, even for longer ozonation of up to 30 min (Figure S7). This was accompanied by a fast production of acidic species, as supported by the noticeable pH decrease. NaMt showed the most attenuated pH decrease, which barely reached 4.67 after 30 min of ozonation (Figure 2a), and a weaker NOF depletion not exceeding 64% (Figure S7a), as compared to the other clay catalysts (>98%) and even to ozone alone (96%). These opposite data, obtained with NaMt and Fe(II)Mt, can be regarded as the limits of the variation range for the catalytic activity of the clay catalysts and, consequently, of the other exchangeable cations.

### 2.4. Effect of Cations on Clay Catalyst Dispersion

The weaker catalytic activity of NaMt contrasts with its higher dispersion in the aqueous media, as reflected by its lower particle size (1608 nm) compared to the other catalysts (Table 2). However, NaMt displayed a particle size (PS) of almost twice larger in the NOF solution than in the pure water (846 nm), with no noticeable difference in the Zeta potential values. This can be explained in terms of partial clay lamellae aggregation induced by repulsive [NOF–clay] interactions. In this case, the NOF adsorption on NaMt, if any, should have a reduced contribution to the heterogenous catalytic process of ozonation due to the basic character of NaMt [57].

The effect of cations is mainly related to their polarizing power (PP), usually and often expressed as (Charge)/(Ionic radius in Å)^2^, being directly proportional to the charge on a cation and inversely proportional to the size of the cation. This factor determines the Lewis acidity strength of the cation and, therefore, its capacity to be neutralized by hydration water molecules that act as Lewis bases and undergo dissociation with proton release [58,59]. This explains why the lowest PP of the Na^+^ cation (1.0) promotes almost no interaction with water molecules and no acidity in aqueous media.

NaMt even induced an alkaline initial pH in the solution of 9.40. This pH is higher than the pKa of both ammonium (8.75) and carboxyl groups (6.34), which will bear no charges due to protonation and negative charges due to deprotonation, respectively. As a result, repulsive interactions towards both deprotonated silanols hinder the electrostatic adsorption of NOF on NaMt, unlike on the other clay catalysts. The latter induce acidic pHs below the pKa of R_2_N groups (8.75), thereby promoting ammonium protonation and interaction with the silanols. The silanols still bear negative charges below their pKa values (5.6 for out-of-plane silanols and 8.5 for in-plane silanols) [25,66,67] and above their pH-Zero Charge (pH_PZC_) of around pH 2–4, depending on the silica type [68,69].

### 2.5. Correlation-Induced pH-Cation Polarizing Power

**Figure 4:**
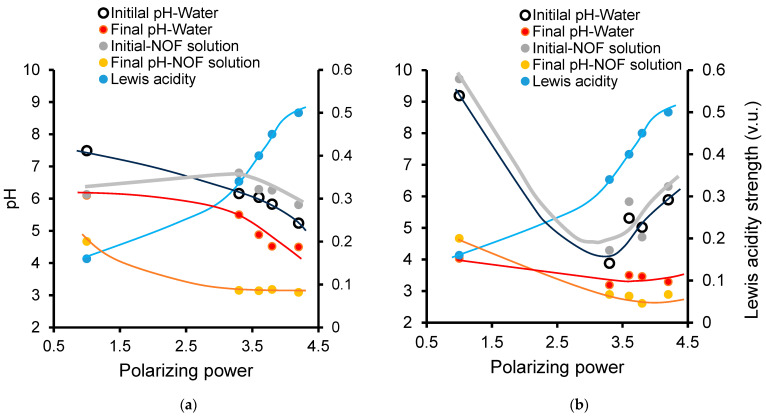
The effect of the cation polarizing power on the pH before and after ozonation in the presence of dissolved cations (**a**) and clay catalysts (**b**). Ozone flow rate: 600 mg/h. Catalyst amount: 2 g/L. Sample volume: 25 mL. NOF concentration: 3.31 × 10^−6^ M. The NOF conversion yield was calculated using the relationship (1 − (A/A_0_)) × 100% and the measurement of the instant/initial peak area ratio (A/A_0_) by HPLC-DAD. The instant pH was measured with variable standard error (±0.2–0.5) during ozonation.

The reverse proportionality between the PP and the induced pH previously observed for dissolved cations (Table 1) appears to be somehow altered in the presence of clay catalysts (Table 2). Indeed, Fe(II)Mt and Ni(II)Mt induced the lowest and highest initial pH levels, respectively, which contrasts with the sequence observed for dissolved cations. In other words, this sequence, i.e., a pH decrease with increasing PP, appears to even be reversed for the initial pH and at least markedly attenuated for the final pH in the clay-catalyzed ozonation of both water and the NOF solution (Figure 4).

Here, the formation of Fe^3+^ as a more acidic exchangeable cation at the expense of Fe^2+^ is more probable given the lower oxidation capacity of Ni^2+^. Nonetheless, the most plausible explanation is that the presence of a negatively charged clay surface (Mt^−^) modifies the sequence of the Lewis and Bronsted acidity strengths of free (dissolved) cations. This must be due to changes in the Lewis basicity of their corresponding counterions, since cation interaction with chloride, for instance, should to differ from that involving Mt^-^ polyanion [58,59]. This finding is of great importance, because it provides evidence that the presence of a clay surface modifies the cation behavior in aqueous media.

### 2.6. Evolution of Catalyst Dispersion During Ozonation

The decrease in the pH during ozonation was found to significantly influence the surface charges of the NOF molecules and the clay lamellae interactions that govern the clay sheet aggregation/dispersion in water. This was illustrated by noticeable changes in the ZP (Figure S8a) and the particle size in aqueous media (Figure S8b), in agreement with the literature [70,71]. These changes are expected to determine the contact surface available for NOF adsorption and degradation.

**Figure 5:**
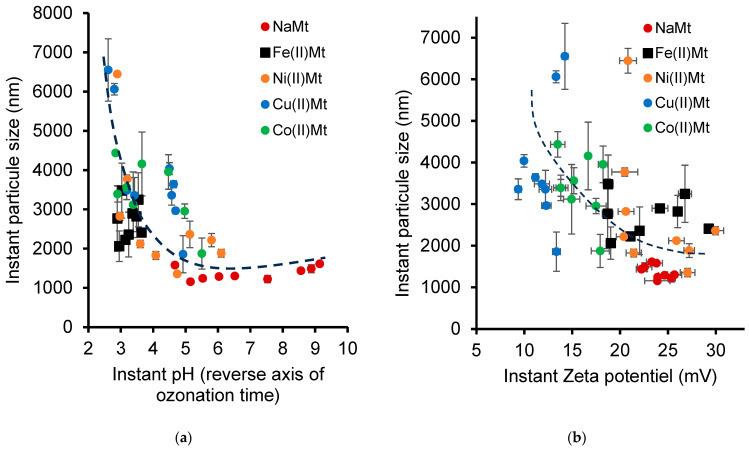
The correlation of the catalyst particle size with changes in the pH (**a**) and the Zeta potential absolute value (**b**) during NOF ozonation. Ozone flow rate: 600 mg/h. Catalyst amount: 50 mg. Sample volume: 25 mL. These experimental data were obtained at different ozonation times from 0 min without ozone to 30 min ozonation, respectively, corresponding to the last mark at the left side and the first mark at the right side of each colored series. The pH was measured with the variable standard error (±0.2–0.5) during ozonation.

There is no clear trend in the effect of the instant pH on the individual evolution of the dispersion of each catalyst, but all the PS values of all of the clay catalysts investigated seem to correlate well with all the measurements of the instant pH induced by NOF ozonation. However, the general tendency is a decrease in the PS of the clay catalyst with the increasing pH that is induced by the ozonation progress (Figure 5a).

This phenomenon is barely visible for NaMt, most likely due to a shading effect of its basic character and to potential [NOF:NaMt] interactions in agreement with our previous statement. However, a much more important finding undoubtedly resides in the general tendency that the particle size increases with the unavoidable pH decrease during ozonation. This phenomenon was markedly more pronounced in the presence of clay catalysts exchanged with transition metal cations. The clay catalyst dispersion appears to strongly depend on the exchangeable cations, which were found to induce specific pH values and Zeta potential values (ZP) (Table 1).

### 2.7. Electrostatic Interaction Changes During Ozonation

The initial pH and Zeta potential (ZP) evolve during ozonation and greatly influence the [clay–clay] and [NOF–clay] interactions and their contributions to the catalytic process. Here also, no clear correlation between the instant PS and ZP can be established for each clay catalyst. This is due essentially to the PP of each exchangeable cation that governs its Lewis acidity and, consequently, the number of hydration molecules and their dissociation into free protons [58,59]. Yet, there exists a vague reverse proportionality between both parameters when correlating all the data for all the catalysts together (Figure 5b). Indeed, the clay particle size appears to somehow increase with the increasing ZP during NOF ozonation, as a result of pH decay.

As previously stated, for all the clay catalysts, a part of the negative charges are permanently compensated by cations [72]. ZP changes arise from silanol deprotonation when the pH is above the pzc for the silica component, typically between pH 2–4 [68,69]. This process is enhanced at pH values higher than the pKa of 5.6 for the “out-of-plane” silanols, and even more so beyond the pKa of 8.5 for the “in-plane” silanols [25,66,67]. Besides, bivalent cations are known to have the capacity to be sandwiched between clay lamellae, inducing stronger interactions that promote flocculation into larger particles and surface contact decay [73]. The consecutive clay aggregation and compaction is expected to affect the clay material efficiency by lowering the accessible adsorption and catalytic sites.

In the presence of the NaMt suspension, the decrease in pH (from 9.14 to 4.64) in the NOF solution after the ozonation process induced a decrease in the particle size from 1612 nm to 1156 nm (Figure S8b). This can be explained by the possible depletion of the repulsive [NOF–clay] interactions due to the progressive protonation of both NOF carboxyl and silanols with the pH decrease [61]. This phenomenon seems to be somehow attenuated in the presence of Fe(II)Mt, which induces an excessive pH decrease (from 3.64 to 2.89). NOF amino group protonation generates positive charges that attract deprotonated silanols. Similar, but less or more pronounced, phenomena were noticed for the other clay-supported metal cations, according to their specific features in aqueous media [74].

### 2.8. Chemical Species Interactions During NOF Adsorption

**Figure 6:**
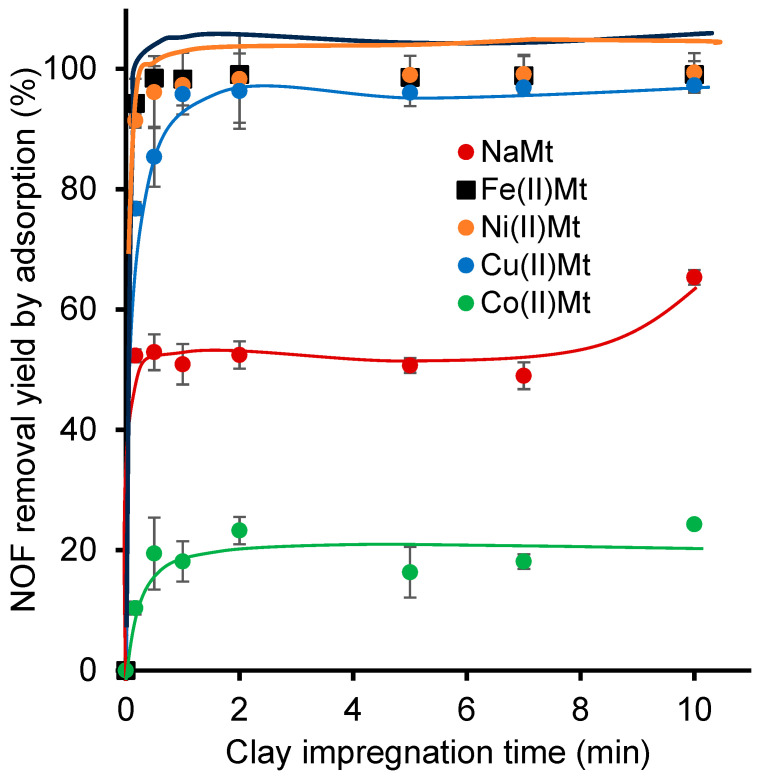
Adsorption isotherms of NOF as expressed by NOF removal yield versus clay adsorbent impregnation time. Sample volume = 25 mL. NOF initial concentration = 3.31 × 10^−6^ M. Catalyst amount = 2 g/L. T = 25 °C. The relative peak area (A/A_0_ calculated as the instant/initial HPLC-DAD peak area ratio of NOF. The adsorption yield was defined as (1 − (A/A_0_)) × 100%, where A is the instant HPLC-DAD peak area and A_0_ the initial HPLC-DAD peak area of NOF solution.

In the absence of ozone, Fe(II)Mt and Ni(II)Mt showed their highest NOF adsorption yields, reaching equilibrium at ca. 99% and 99.4%, respectively, after only 1 min of contact time (Figure 6). Fe(II)Mt showed a slightly slower NOF adsorption, reaching surface saturation at approximately 97–97% after 2 min of contact time. Deeper insights into the adsorption mechanisms were achieved by applying kinetic models, which revealed that the process occurs mainly via the pseudo-second order, based on the closest correlation coefficient values (R^2^) to unity (Table 3).

This result provides confirmation that metals can retain antibiotics and clear evidence that NOF adsorbs preponderantly via chemical interactions with the clay catalyst surface, most likely through ion-exchange. Such an adsorption process should involve the direct interaction of the negatively charged clay surface with the positive charges of the amino groups of NOF. These positive charges are induced by the protonation at the pH range induced by clay suspensions below the pKa of the amino group, this process being more attenuated for NaMt (Table 2).

Hence, electrostatic adsorption seems to prevail, even though NOF adsorption occurs through different functional groups [75]. This turned out to be a common feature of FQ adsorption on cation-exchanged montmorillonite [76]. Adsorption should also involve chelation through Lewis acid–base interactions between cations and NOF moieties bearing non-binding electron pairs. Reportedly, ciprofloxacin (CIP) can chelate metals ions via oxygens of carbonyl or carboxylic groups, and similar interactions were observed in the presence of a humic substance [77]. Coordination interaction was also reported between some FQs and Fe^3+^ and Cu^2+^ cations [31]. Coordination reactions between three other FQs and two ions Fe(III) and Cu(II) were observed with UV–Vis at different pH levels in previous work [31].

Given the narrow interdependence of the ZP and the PS with the amount of dispersed NOF molecules and that the induced pH evolves during ozonation, adsorption is also expected to evolve over time and significantly influence the global process kinetics. Hence, the mere presence of clay catalysts was found to accelerate the ozonation process due to the adsorption contribution, even before the complete dissolution of the ozone in the aqueous medium [61].

The potential occurrence of ternary [M^n+^:Mt:NOF] interactions is supported by composing binary ones. The theoretical modeling of such interactions is based on metal cation capture/release by the following: 1. ion-exchange by the permanent charges of the octahedral Al layer, partially substituted by Mg^2+^ and/or Fe^2+^ ions; 2. the pH-dependent charges of the silanol groups; 3. the Lewis acid–base chelation of excess metal cation by non-binding electron pairs belonging the lattice oxygen atoms; 4. pH-dependent charges of NOF carboxyl groups; 5. the Lewis acid–base chelation of excess metal cations by the non-binding electron pairs of the N-atoms of NOF; 6. NOF adsorption on the clay surface by cation exchange through protonated NOF amino groups at a low pH, 7. repulsive interaction between deprotonated clay silanols and NOF carboxyls at a high pH; 8. H-bridges of clay silanols and aluminols, mainly with NOF carboxyls; 9. hydrophobic interactions between NOF molecules and -Si-O-Si-rich islands. In the presence of ozone, quaternary [M^n+^:Mt:NOF:O_3_] interactions are also possible if one assumes, at least, that the mere presence of a clay surface favors ozone adsorption, mainly via ozone molecule polarization and electrostatic interactions [22,23,53,56].

### 2.9. Clay Dispersion Effect on Toxicity

**Figure 7:**
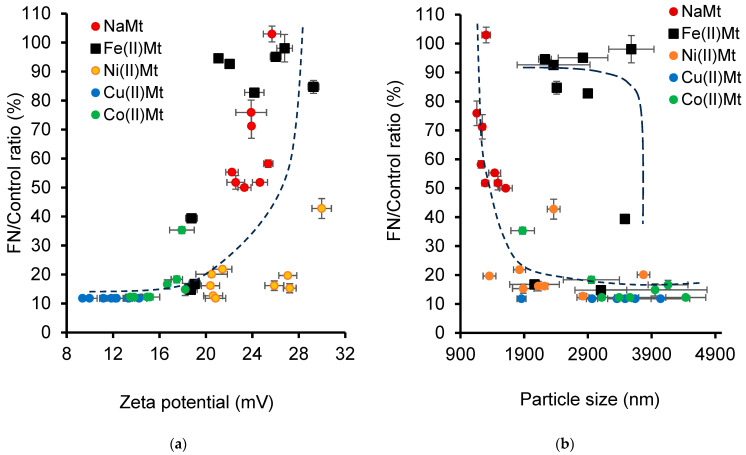
The effect of the Zeta potential absolute value (**a**) and particle size (**b**) on the frond number of *Lemna minor*. The frond number (FN)/Control ratio was defined as being the (measured FN/Control FN ratio) × 100%. The control consisted of an average fresh weight of 98 mg of the plant and 67 fronds in normal growth conditions in an SIS medium, not exposed to ozonized NOF mixtures.

The pH evolution over time should be a key-factor in governing the surface charge and clay dispersion, which, in turn, control their very catalytic activity, ozonation process advancement, product distribution, and consequently, the toxicity of the ozonized NOF mixture. In this regard, the Zeta potential and particle size measurements may provide valuable information on the toxicity of heavy metals and organic pollutants [78,79,80]. This narrow interdependence between all these factors can be illustrated by an increasing tendency in the FN/Control ratio with increasing Zeta potential (Figure 7a).

Given that the FN is a primary indicator used to assess the phytotoxic effects of antibiotics and heavy metals [78,79], a first plausible explanation should be that NaMt and Fe(II)Mt contain less toxic and even more nutritive cations towards *L. minor* as compared to the other clay catalysts. Nonetheless, these beneficial features of NaMt and Fe(II)Mt must be combined with those of their lower instant particle sizes with an increasing instant ZP (Figure 5b). As expected, such a data combination gave rise to a decreasing tendency of the FN/Control ratio with the increasing particle size (Figure 7b).

A similar but weaker detrimental effect of the large particle size was noticed for Fe(II)Mt at a high FN/Control ratio due to its lower toxicity and higher nutritive properties as compared to the other exchangeable cations. Thus, both Na^+^ and Fe^2+^ promote clay dispersion at a specific pH and via different mechanisms, leading to increased clay activity, high NOF conversion, and reduced toxicity [81]. The basicity of Na^+^ enhances silanol deprotonation, repulsive forces, and clay particle dispersion. Fe^2+^ hydration induces Bronsted acidity via hydrolysis into free protons and additional negative charges on the [Fe^2+^(H_2_O)_(n−1)_OH-] cation on the clay surface and reduces its sandwiching effect between clay lamellae [73].

### 2.10. Cation Intrinsic Toxicity on Frond Number

**Figure 8:**
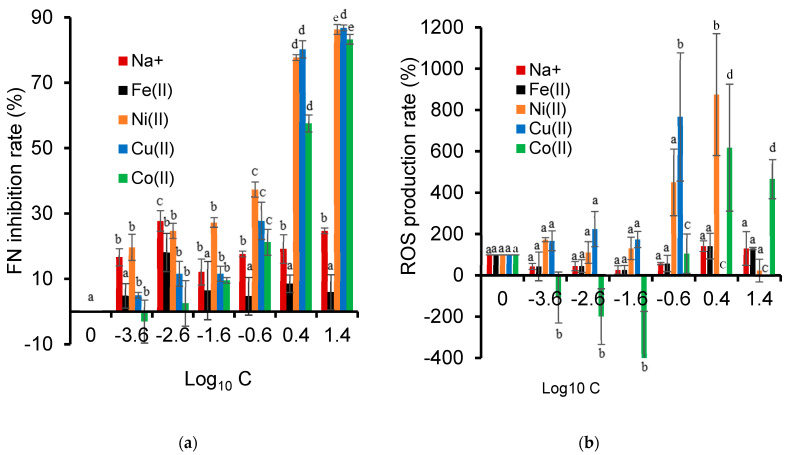
The concentration effect of cations alone dissolved in water on their toxicity to *L. minor* as expressed in terms of the inhibition of frond number growth (**a**) and the ROS relative amount (**b**) as reported to the blank sample (0 mg/L). These toxicity parameters were measured after 7 days of exposure in an SIS medium containing cation solutions at concentrations ranging from 0.00025 to 25 mg/L (the solutions were prepared by the dilution of a stock solution of 25.10 mg/L ± 0.1). The results are expressed as percentages relative to the control sample (SIS medium). Triplicate measurements were achieved for all the concentrations considered, including the metal-free control samples. The control consisted of plants grown in an SIS medium, with an average fresh weight of 98 mg and 67 fronds. Certain negative values indicate an increase in growth (**a**) and a decrease in the ROS level (**b**) compared to the control group (0 mg/L). The different symbols used (a, b, c, d and e) account for different data significances at *p* < 0.05. Data with similar significance are marked with similar symbols in the same group.

Here, the toxicity towards *L. minor* unavoidably included that of the metal cations, which was reflected by the increasing frond number (FN) inhibition rate and the production of oxygen reactive species (ROS) with the increasing cation concentration, expressed in terms of Log_10_ C (Figure 8). This phenomenon was a common behavior for all the metal cations but was increasingly more pronounced for Co^2+^, Ni^2+^, and Cu^2+^ with the increasing concentration.

It is worth mentioning that Co^2+^ displayed the lowest FN inhibition rate and ROS production at a low concentration of around 25 × 10^−4^ ppm. At this concentration, Co^2+^ is known to act as a micronutrient that stimulates plant growth. Co^2+^ at concentrations higher than 0.059 ppm was already found to inhibit *L. minor* growth by inhibiting iron transport from the roots to shoots without affecting iron uptake [82]. Other metal cations, such as Cu^2+^, can exhibit toxicity towards *Lemna minor*, even at low concentrations, by favoring the activity of the antioxidant enzymes catalase and guaiacol peroxidase [83]. Plants are known to raise the activity of their antioxidant defense systems in response to oxidative stress that reduces the ROS levels.

ROS formation is one of the primary drivers of toxicity when dealing with genotoxicity [84,85] or phytotoxicity assessment [86]. At a high cation concentration, the ROS disappearance is often attributed to severe cellular damage and the dysfunction/destruction of ROS-producing organelles, such as chloroplasts, mitochondria, and peroxisomes [87]. Other living species, such as the microalga *Raphidocelis subcapitata*, showed increased ROS production after exposure to Co^2+^ (0.5 mg/L), as well as a decay in the fluorescence of chlorophyll *a* (0.10 mg/L). Plants of the same species rather showed a decrease in ROS production after Ni^2+^ exposure due to an activation of their antioxidant mechanisms [88].

The observed toxic responses to Na^+^ concentrations involved only growth inhibition. Salt excesses above certain thresholds can lead to physiological disruption in *Lemna minor* and *Pseudokirchneriella subcapitata*, and no ROS involvement and no reduction in chlorophyll was noticed [89]. Indeed, Na^+^ toxicity appears to only arise from osmotic stress caused by salt excess [48]. Depending on the dose and type of metals, the results obtained demonstrate that redox active (Co, Cr, Fe, and Cu) and non-redox active (Ni and Zn) metals can have an impact on *L. minor* plants via different toxicity mechanisms.

This intrinsic toxicity of metal cations that inhibits Lemna minor growth, particularly at relatively high concentrations beyond admissible levels, was somehow expected. Certain metal cations can compete with other essential micronutrients at physiologically important chelating sites, leading to functional deficiencies that affect living species’ metabolisms [39]. A comparison with cation-exchanged clay materials revealed a higher intrinsic toxicity of free dissolved cations for *L. minor* as compared to their clay-supported counterparts [81]. This is mainly due to a lower availability in the dispersed form in the bulk aqueous media. Negatively charged soil surfaces, including cationic clay-based materials, such as smectites, are well known to control pollution by metals through both cation-exchange and chelating processes by atoms acting as Lewis bases and exhibiting available non-binding electron pairs.

### 2.11. Effect on Photosynthetic Efficiency

The exposure of *L. minor* to cations affected the photosynthetic efficiency and electron transfer in PSII. Cation concentrations of 0.25–25 μg/L showed no marked effect on the pigment production (Figure S9). The pH set in the SIS medium or induced by the addition of reaction mixtures resulting from cation- and clay-catalyzed NOF ozonation and clay’s intrinsic effect on FN are separate key-factors in ecotoxicity changes (Figures S10–S12).

**Figure 9:**
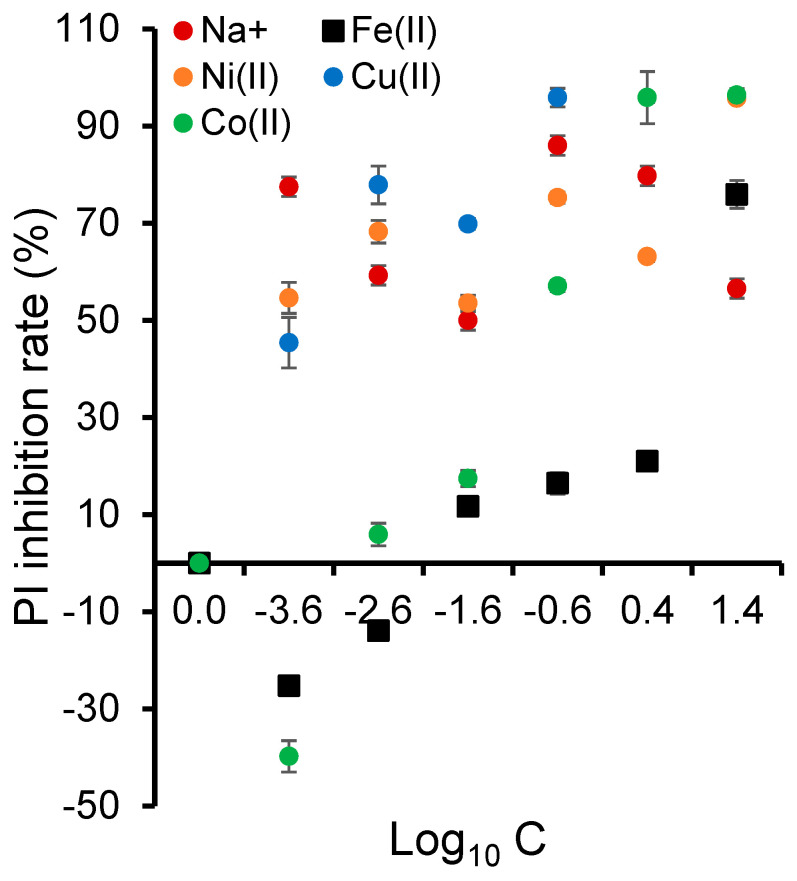
The change in the inhibition rate of the Performance Index, PI_ABS_ (% of the control). *L. minor* plants were exposed for 7 days to different cation concentrations (mg/L). Triplicate measurements were achieved, including the metal-free control sample. The control sample consisted of plants grown in an SIS medium, with an average fresh weight of 96 mg and 66 fronds not exposed to ozonized mixtures.

Deeper insights into fluorescence kinetics revealed a decay in the relative fluorescence intensity from ca. 3.5 down to approximately 2.5 with the increasing cation concentration up to 25 mg/L (Figure S13), indicating that metal cations interfere with the photosynthetic electron transport and energy transfer processes in the thylakoid membrane. In addition, the photosynthetic parameters calculated herein, i.e., the inhibition rate of the Performance Index, PI_ABS_ (Figure 9), the maximum PSII quantum yield, Fv/Fm (Figure S14a), and the variable fluorescence relative to quinone A reduction, (Vj) (Figure S14b), showed noticeable changes, particularly for high concentrations of all the cations. These results, obtained on the photosynthetic response to the metal concentration, confirm the PSII multi-site toxicity pattern across various transitional metal cations.

Among these parameters, PI_ABS_ is particularly interesting, reflecting possible structural and functional variations associated with the PSII complex and offering a holistic assessment of plant vitality [90]. A 55% decrease of PI_ABS_ was observed for copper, sodium, and nickel for the lowest concentration tested of 0.00025 mg/L (Log_10_ C = −3.6). This indicates that transitional metal cations are highly toxic to the photosynthetic performance, even at low concentrations. Under similar conditions, the minimum effects of Fe^2+^ and Co^2+^ suggest the existence of specific physiological mechanisms that allow plants to tolerate or assimilate these cations more effectively. Interestingly, exposure to Fe^2+^ and Co^2+^ had only negative effects on the PI_ABS_ values compared to the control sample at high tested concentrations, suggesting differential toxicity mechanisms for transitional metal cations. The decrease of PI_ABS_ confirmed the inhibitory impact of cations such as Pd^2+^ and Cd^2+^ on PSII photochemistry and electron transport in the thylakoid membrane [91,92,93].

Reduced Fv/Fm values were already reported for *L. minor* plants exposed to different concentrations of metal cations, such as Co, Cs, Mn, Ni, and Zn [27]. In addition, the release of Cu^2+^ cations from CuO nanoparticles was also found to inactivate the PSII reaction center (RC) in *Lemna gibba*, primarily through photoinhibitory processes [91]. Cd^2+^ cation accumulation on green algae, *Chlamydomonas reinhardtii*, caused the inactivation of PSII RC through cation interference with the electron flow required for the light reactions of photosynthesis [92]. Metal cations also exhibit catalytic activity through the formation of reactive free radicals, contributing to additional oxidative stress in plant cells [94]. In this study, the changes in the photosynthetic efficiency observed in the presence of cations and clay catalysts was an accurate indicator of how the [clay–metal–organic substrate] interactions affect the toxicity toward the *L. minor* plants.

In normal cellular metabolism, the electron transfer through electron transport chains occurs in the chloroplasts (Photosystem I and II), mitochondria (Complexes I and III), and peroxisomes [95]. Random electron leaks first generate ROS and superoxide anions is the first ROS formed by reacting with molecular oxygen (O_2_) [96]. Other simultaneous processes also take place [8,97,98,99,100]. Transition metal cations are known to convert H_2_O_2_ into highly toxic hydroxyl radicals (OH·) that cannot be neutralized by plant enzymes [101,102]. This induces a negative impact on PSII and, mainly, on chlorophyll *a* degradation that affects carbon dioxide fixation and cellular structures and metabolisms. [103,104,105]. Besides, metal cations also enhance the activity of chlorophyllase enzyme and can replace Mg^2+^ in the porphyrin ring of the chlorophyll molecules [106].

### 2.12. Toxicity of Cation-Catalyzed Ozonation

**Figure 10:**
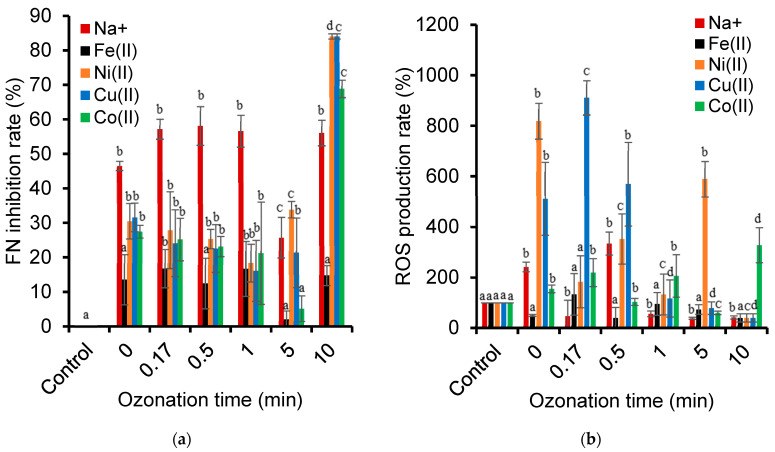
Toxicity of ozonized NOF solution in presence of metal cations, assessed as frond number inhibition rate (**a**) and ROS production (**b**) of *Lemna minor* after 7 days of exposure. Triplicate measurements were achieved, including the metal-free control sample. The control sample consisted of plants grown in an SIS medium, with an average fresh weight of 96 mg and 66 fronds that were not exposed to the ozonized mixtures. The different symbols used (a, b, c and d) account for different data significances at *p* < 0.05. Data with similar significance are marked with similar symbols in the same group.

A quick overview of frond number measurements during cation-catalyzed ozonation revealed that the nature of the cations has specific effects (Figure 10a). After an ozonation time of up to 10 min, the Na^+^-containing reaction mixtures showed the highest FN inhibition rate (ca. 46–58%), while the Fe^2+^ cation induced the lowest levels of approximately 13–17%. The Ni^2+^, Cu^2+^, and, to a lesser extent, the Co^2+^ cation showed a marked toxicity increase after 10 min of ozonation. In agreement with previous statements, this was only accompanied by weak ROS production by all the cations. This ROS production thoroughly depleted below the natural level for the control sample after 1 min of Na^+^-catalyzed ozonation (Figure 10b).

In the meantime, decreases in the chl *a/b* ratio, but barely detectable for Fe^2+^ and Co^2+^, were noticed once ozonation was triggered (Figure S15). After an ozonation time of 10 min in the presence of Co^2+^ and Fe^2+^, the NOF conversion yield increased up to 94.9% and 94.5%, respectively, without inducing significant ROS production. The Fe^2+^ cation also enabled the total mineralization of fluoroquinolone and reduced ecotoxicity [107]. The fact that organic substrates such as ciprofloxacin (CIP) can chelate metal cations through their Lewis base sites is a plausible explanation of the toxicity mitigation, as already reported for *E. aeruginosa* [77].

Notwithstanding that longer contact times with metals are known to enhance toxicity, the mere presence of NOF molecules should reduce the cation mobility/release in the bulk solution. In addition, the acidic conditions induced by cation-catalyzed ozonation unavoidably favor cation exchange with negatively charged NOF molecules [22]. NOF can also bind to divalent metal ions via chelation between the cation and the 4-oxo and the carboxyl group adjacent to the organic substrates, which may result in changes in their toxicity, with possible adverse effects on fauna and flora induced by FQ–metal complexes [32]. There, both cation adsorption via ion-exchange and chelation, along with ozonation, are expected to mitigate not only the toxicity of the cation but also those of the residual and progressively depleting NOF molecules and oxidized derivatives that should also bear additional oxygen atoms and non-binding electron pairs. This is a plausible explanation of the decay in ROS production as the ozonation process progresse

## 3. Methods and Materials

### 3.1. Clay Material’s Preparation and Dispersion in Aqueous Suspension

A montmorillonite-rich sample (NaMt) (cation exchange capacity = 99 ± 3 meq/100 g; specific surface area = 34 m^2^/g, silica/alumina weight ratio = 1.58; montmorillonite content = 84 wt%) was prepared from a crude bentonite supplied by Sigma Aldrich (Burlington, MA, USA) according to a procedure described previously [71,81]. Other ion-exchanged counterparts (Fe(II)Mt, Ni(II)Mt, Co(II)Mt, and Cu(II)Mt) resulted from the ion exchange of 9 g of dry NaMt powder in 6 g/L of each corresponding aqueous solution of metal salts.

FeCl_2_.4H_2_O, NiSO_4_.6H_2_O, CoCl_2_.7H_2_O, supplied by Fisher Scientific, Pittsburgh, PA, USA (99% purity), and CuSO_4_.5H_2_O (99.995% purity, Sigma Aldrich, Burlington, MA, USA), in Nanopure water. The mixture was stirred at 80 °C for 7 h and cooled overnight at room temperature (RT). The resulting suspension was repeatedly washed with distilled water, centrifuged, dried at RT and characterized by X-ray diffraction (D8 Advance Brücker, Madison, WI, USA, CuKα at 1.54051 Å). All the clay catalysts showed sharp 001 X-ray diffraction lines (Figure S1) and perfectly parallel clay sheet layouts. Na^+^ exchange induced a shift of the 001 line towards lower 2-Theta due to an enlargement of the interlayer space [61,81]. Additional characterization by Fourier Transform Infrared spectroscopy (Thermo Scientific, Nicolet 6700 instrument, Madison, WI, USA) revealed characteristic vibration bands at 3626 cm−1 (Al–OH stretching), 985 cm^−1^ (Si–O stretching), 908 cm^−1^ (Al–OH bending), and 796 cm^−1^ (Si–O stretching), as others already reported [81,108].

The clay material dispersion in aqueous media was evaluated through the measurement of the particle size (PS) and zeta potential (ZP), which provides valuable data on the pH’s effect on the particle suspension [109]. This was achieved with 50 mg of clay powder, previously dispersed in 25 mL of Nanopure water or aqueous NOF solution, after each catalytic ozonation test. The two analyses were carried out using a Malvern Zetasizer device Ultra Red Label (Malvern Panalytical, Malvern, UK) at 25 °C, while the data acquisition was achieved with a Zeta potential analyzer, Ver.5.68 software, and plastic cuvettes (DTS1070, Malvern instruments LTd, Worcestershire, UK).

### 3.2. Adsorption Tests

The NOF powder (NOF, Lot No LRAD2292), purchased from Sigma Aldrich, was dissolved in nanopure water at specific concentrations, considering the solubility of NOF at ambient conditions (3.31 × 10^−6^ M). Adsorption experiments were carried out by exposing 50 mg of dry clay mineral to 25 mL of NOF solution at an intrinsic pH under vigorous stirring at RT in a 50 mL Erlenmeyer flask. The samples were analyzed immediately after filtration through 0.45 mm membrane. Insights in NOF adsorption kinetics were achieved through measurements of the instant and equilibrium amounts of the adsorbate (***qt*** and ***qe***, respectively) for each sample. The pseudo-first-order and pseudo-second-order models were then plotted as functions of time using Equations (1) and (2), respectively:

(1) $$Log\;\left(q_{e}-q_{t}\right)=Log\left(q_{e}\right)-\frac{K_{1}}{2303}t$$

(2) $$\frac{t}{q_{t}}=\frac{1}{K_{2}\times {q_{e}}^{2}}+\frac{t}{q_{e}}t$$

Allowed calculating *K_1_* and *K_2_*, the rate constants of pseudo-order 1 and 2, respectively.

### 3.3. Ozonation Tests

Ozonation tests were performed by ozone bubbling (600 mg/h) in a series of glass tubes (25 mm × 200 mm) containing 25 mL samples of NOF solution (3.31 × 10^−6^ M), either without a catalyst, in the presence of 50 mg of a dry clay catalyst, or in the presence of Fe(II), Ni(II), Cu(II), and Co(II) cations at a 2.5 × 10^−3^ mg/L concentration to evaluate their effect at RT.

The concentration of dissolved ozone was determined using the iodometric method [110]. Fully described procedures have been added in the Supporting Information File (SI). For this purpose, the A2Z-AQUA-6 portable ozone generator (A2Z Ozone Inc., Louisville, KY, USA) was pre-calibrated using another portable ozone generator (A2Z-MP 8000, Louisville, KY, USA) equipped with a built-in internal air compressor and buttons for adjusting the ozone concentration and bubbling time. This pre-calibration also confirmed the value of the gas ozone throughput of 600 mg/h provided by the original factory calibration and allowed us to determine the time evolution of the O_3_ concentration in water. The results support the assumption of an pseudo-linear relationship between the bubbling time and the dissolved ozone concentration during the initial phase (10–15 min) [61]. Regardless of the ozone concentration in the carrier gas or the operational conditions (600 mg/h versus 8000 mg/h), the dissolved ozone concentration remained limited to approximately 0.4 mmol/L, due to physicochemical constraints [67]. This value is much lower than the reported 20.8 mmol/L in water at 0 °C [111] or 11.9 mmol/L in water at 20 °C [23]. Even under optimal conditions, the solubility of ozone in water remains limited [112,113]. The resulting O_3_/NOF molar ratio of ca. 121 ensured a sufficient excess of ozone after 10 min ozonation.

The possible release of metal cations upon clay mineral leaching in NOF solution and during ozonation was investigated through Inductively Coupled Plasma Optical Emission spectroscopy (ICP-OES) using an Agilent Technologies-5100 device (Santa Clara, CA, USA) with an axial plasma configuration and a concentric quartz nebulizer under specific conditions (Table S1). For the digestion method, 2 mL samples of the ozonized mixture were mixed into 2 mL of HNO_3_ (Fisher Scientific, purity 70%), and the glass tubes were heated in a sand bath at 90 °C for 1 h. After digestion, each sample was acidified with HNO_3_ to obtain a final concentration of 5% HNO_3_ prior to the analysis.

After ozonation and catalyst removal by centrifugation and filtration, the supernatant was analyzed through UV–Vis spectrophotometry (Agilent-Cary 60 instrument, Santa Clara, CA, USA, 1 cm quartz cell) and High-Performance Liquid Chromatography coupled to an ultraviolet-visible detector (HPLC-DAD), Agilent Technologies model 1290 equipment under specific conditions (Table S2). The ozonation process was investigated at either the initial intrinsic or at the initial pH of the reaction mixture, which was adjusted by adding a few drops of 0.1 M sodium hydroxide (NaOH) or hydrochloric acid (HCl). The pH was measured before and after ozonation using an Accumet^®^model 15 pH-meter (Fisher Scientific, Pittsburgh, PA, USA), with an accuracy of ±0.01 units.

### 3.4. Toxicity Tests on Lemna Minor

*Lemna minor* was used as a bioindicator and was cultivated in a nourishing medium, previously prepared according to the Swedish Institute for Standards (SIS), containing the following (mg/L): NaNO_3_, 85; KH_2_PO_4_, 13.4; MgSO_4_.7H_2_O, 75; CaCl_2_.H_2_O, 36; Na_2_CO_3_, 20; H_3_BO_3_, 1; MnCl_2_.4H_2_O, 0.20; Na_2_MoO_4_.5H_2_O, 0.010; ZnSO_4_.7H_2_O, 0.050; CuSO_4_.5H_2_O, 0.005; Co(NO_3_)_2_.6H_2_O, 0.010; FeCl_3_.6H_2_O, 0.84; Na_2_-EDTA.2H_2_O, 1.4; buffer MOPS, 490. The medium pH was adjusted to 6.5 ± 0.2, and the resulting SIS medium was sterilized at 121 °C under vacuum in a steam sterilizer, model LSS 275, for 30 min. The stock culture of *L. minor* specimens was maintained under standardized growth conditions in cotton-sealed Erlenmeyer flasks to reduce evaporation and prevent contamination. The plant cultivation was achieved under 16 h of light exposure (a light intensity of 100 ± 10 µmol of photons m^−2^s^−1^ with white, fluorescent lamps) and 8 h of dark cycles for 7 days at 24 °C ± 2 °C and a relative humidity of 60 ± 5%.

In a first step, the intrinsic toxicity of the metal cations (Na^+^, Fe^2+^, Ni^2+^, Cu^2+^, and Co^2+^) towards *Lemna minor* was investigated by placing, healthy, green, three-fronded specimens of *L. minor* in 50 mL Erlenmeyer flasks containing different cation concentrations (2.5 × 10^−4^–25 mg/L), prepared in nanopure water using the same metal salts as for the clay catalyst preparation, for 7 days. These initial concentrations were selected to cover the full range of natural to highly contaminated conditions [114,115]. In the second step, the toxicities of the cations, both alone and adsorbed on montmorillonite, were evaluated in the supernatant of the NOF solutions, before and after ozonation. For this purpose, in addition to the toxicity criteria already investigated in previous work [81,108], such as the FN, chlorophyll content, and relative amount of reactive oxygen species (ROS), the photosynthesis efficiency was measured. The methods used for assessing ROS and other parameters are described elsewhere [81,93]. This provides an insight into the impact of metal cations on the toxicity of oxidative NOF degradation. The number of fronds and fresh weight were estimated in each flask after 7 days of exposure and compared to control samples consisting of plants in an SIS medium non-exposed to ozonation mixtures. The growth rate inhibition was then calculated in relation to the control using Equation (3), where µ is the specific growth rate for the FN.

(3) $$\%\;inhibition=100\times \left(1-\frac{\;u_{sample}}{u_{control}}\right)$$

The photosynthetic efficiency of *L. minor* was determined by using the chlorophyll fluorimeter Handy-PEA (Plant Efficiency Analyser, Hansatech Instruments Ltd., King’s Lynn, UK), which is designed for the rapid and high-resolution measurement of chlorophyll fluorescence from Photosystem II (PSII). The chlorophyll fluorescence kinetics provide key insights into PSII functionality under varying conditions. Prior to measurements, *L. minor* fronds were submitted to 30 min of dark adaptation to quench fluorescence and favor full oxidation of the electron transport chain. Further, the plants were exposed to a light pulse (intensity of 3000 µmol of photons m^−2^s^−1^, maximal emission at 650 nm) during one second.

From the different phases of the fluorescence kinetic, several parameters can be estimated reflecting the photosynthetic electron transport dynamics. However, this study was restricted to the Performance Index (PI_ABS_), which is a holistic metric quantifying the efficiency of the photosynthetic biological system by integrating multiple functional aspects of PSII energy fluxes and offering a sensitive diagnostic tool for plant stress physiology [93,116,117]. The parameter PI_ABS_ was calculated as PI_ABS_ = RC/ABS × φPo/(1 − φPo) × ψEo/(1 − ψEo), where 1. RC/ABS indicates the density of active reaction centers (RC) per energy dissipation from chlorophyll antenna. 2. φPo, estimated as the quantum yield of primary photochemistry, reflects the efficiency of light energy conversion into redox energy, φPo = 1 − Fo/Fm. The Fo represents the fluorescence intensity at 20 µs, and Fm is the maximum fluorescence intensity. 3. ψEo is the efficiency of electron transport beyond the quinone A (Q_A_) in the PSII, derived from ψEo = 1 − [(Fj − Fo)/(Fm − Fo)]. The Fj represents the fluorescence intensity at 2 ms. The other parameters of the photosynthetic system efficiency were also assessed using their corresponding equations and summarized in the Supporting Information File (SI).

For toxicity tests, the means and corresponding standard deviations were determined for triplicate measurements (Tables S3 and S4). The differences between the treated and control samples were tested using a one-way ANOVA followed by a Tukey’s post-hoc test for *p* < 0.05, using OriginPro 2025 software version 10.2.0.188.

## 4. Conclusions

This research provides another contribution for a better understanding of the role of clay-containing media in nature self-regeneration, which allows the tailoring of nature-inspired and clay-based water treatments. The results obtained allow us to conclude that a judicious compromise between acidic media and a small clay particle size is favorable for the degradation of norfloxacin. Excessively acidic aquatic media in contact with decaying vegetation and soluble humus derivatives are expected to mitigate the contact surface and catalytic activity of the clay suspension, leading to exchangeable cation loss, the low degradation of organic pollutants, and increased ecotoxicity. Conversely, weakly acidic-to-neutral and even alkaline media are also detrimental to pollutant adsorption and oxidative degradation, and thus to biodiversity. Different metal cations appear to strongly influence this compromise and the toxicity towards the vegetal species. Metal cation interactions with organic pollutants reciprocally modify their respective intrinsic toxicities. Cation capture by norfloxacin mainly involves ion-exchange and chelation and appears to contribute, with ozonation, to attenuating the global toxicity of the ozonized aqueous media. The presence of Na^+^ and Fe^2+^ cations improves the clay dispersion and reduces the toxicity in oxidized, antibiotic-contaminated clay media. The presence of a clay surface modifies the cation behavior in aqueous media, and vice versa. In addition, the photosynthetic organism *L. minor* acted as a reliable and sensitive indicator of the toxicity impact of [clay–metal–organic substrate] interactions, depending on the concentration and type of metal cations. The combined toxicity of metal cations and organic pollutants on the photosynthetic efficiency of vegetal living species still remains to be elucidated. Therefore, these results provide valuable information on the impact of organic pollutant oxidation on oxidative water treatments and on aquatic media.

## Figures and Tables

**Table 2:** Initial physicochemical features of the aqueous clay suspensions before ozonation.

Clay Catalyst	Aqueous Medium	ZP (mV) ^a^	Standard Deviation	PS ^b^ (nm)	Standard Deviation	Initial Charges				pH		
						NOF ^c^		Silanols ^d^				
						R_2_N	-CO_2_^−^	Out	In	Initial ^e^	Final ^f^	Diff. ^g^
None	NOF_(aq)_*					++	-	X	X	5.28	2.44	2.84
NaMt	Water	−23.45	1.663	846	25.65			- - -	- - -	9.19	4.03	5.16
	NOF_(aq)_*	−23.36	0.5761	1608	101.7	+	- - -	- - -	- - -	9.72	4.67	5.05
Fe(II)Mt	Water	−30.5	0.7215	2515	232.6			+/-	+/-	3.88	3.19	0.69
	NOF_(aq)_*	−28.71	0.876	2409	702.4	+++	-	+/-	+/-	4.29	2.89	1.4
Co(II)Mt	Water	−19.54	0.9465	1972	397.5			- -	-	5.31	3.50	1.81
	NOF_(aq)_*	−17.93	1.059	1875	184	+++	- -	- -	-	5.83	2.84	2.99
Ni(II)Mt	Water	−23.54	0.3172	2147	338.5			- - -	- -	5.89	3.29	2.6
	NOF_(aq)_*	−29.97	0.8394	2362	98.46	+++	- - -	- -	-	6.31	2.89	3.42
Cu(II)Mt	Water	−14.95	0.4041	5068	472.5			- -	-	5.02	3.46	1.56
	NOF_(aq)_*	−13.36	0.4912	1858	69.51	++	- -	- -	-	4.71	2.61	2.1

* NOF_(aq)_*: solution concentration: 3.31 × 10^−6^ M; ^a^ ZP: Zeta potential; ^b^ PS: particle size; ^c^ arbitrary charge scale based on pKa values of 8.75 and 6.34 for ammonium and carboxyl groups of NOF, respectively: 1. +++ or ---: >50%, 2. ++ or --: ~50%, 3. + or -: <50%; ^d^ arbitrary charge scale based on pKa values of 5.6 and 8.5 for in-plane (In) and out-of-plane (Out) silanols, respectively: 1. ---: >50%, 2. ++ or --: ~50%, 3. + or +/-: <50% with possible rise of positive charges around pH 2–4; ^e^ initial pH before ozonation; ^f^ final pH after 30 min of ozonation. All pH values were measured with an average standard deviation of 0.01; ^g^ Diff.: pH difference between the initial and final values during ozonation. X: feature not applicable.

**Table 3:** Adsorption kinetics constants and correlation coefficients, as assessed with HPLC-DAD data.

Clay Material	Pseudo-First Order		Pseudo-Second Order	
	K_1_	R_1_ ^2^	K_2_	R_2_ ^2^
NaMt	1.165	0.486	13.944	0.966
Fe(II)Mt	4.048	0.205	444.532	0.999
Co(II)Mt	2.851	0.386	32.303	0.949
Ni(II)Mt	3.122	0.544	123.296	0.999
Cu(II)Mt	2.689	0.574	58.055	0.999

R_1_^2^ and R_2_^2^ are correlation factors for the two different mathematical models.

## Data Availability

Data will be made available on request to the corresponding author.

## Supplementary Materials

The following supporting information can be downloaded at https://www.mdpi.com/article/10.3390/ijms26094347/s1.
