# Supplementary material for: Norfloxacin Oxidative Degradation and Toxicity in Aqueous Media: Reciprocal Effects of Acidity Evolution on Metal Cations and Clay Catalyst Dispersion
Source: Int J Mol Sci. 2025 May 2;26(9):4347. doi: 10.3390/ijms26094347 (PMC12072835; doi:10.3390/ijms26094347)
Supplement: Supplementary file 1 [file ijms-26-04347-s001.zip › ijms-3583451-supplementary.pdf]

Reciprocal effects of acidity evolution on metal cations and clay catalyst dispersion in Norfloxacin oxidative degradation and toxicity in aqueous media

Roumaissa Djidja et al.

Supporting information

1. XRD patterns of bentonites and exchanged montmorillonites

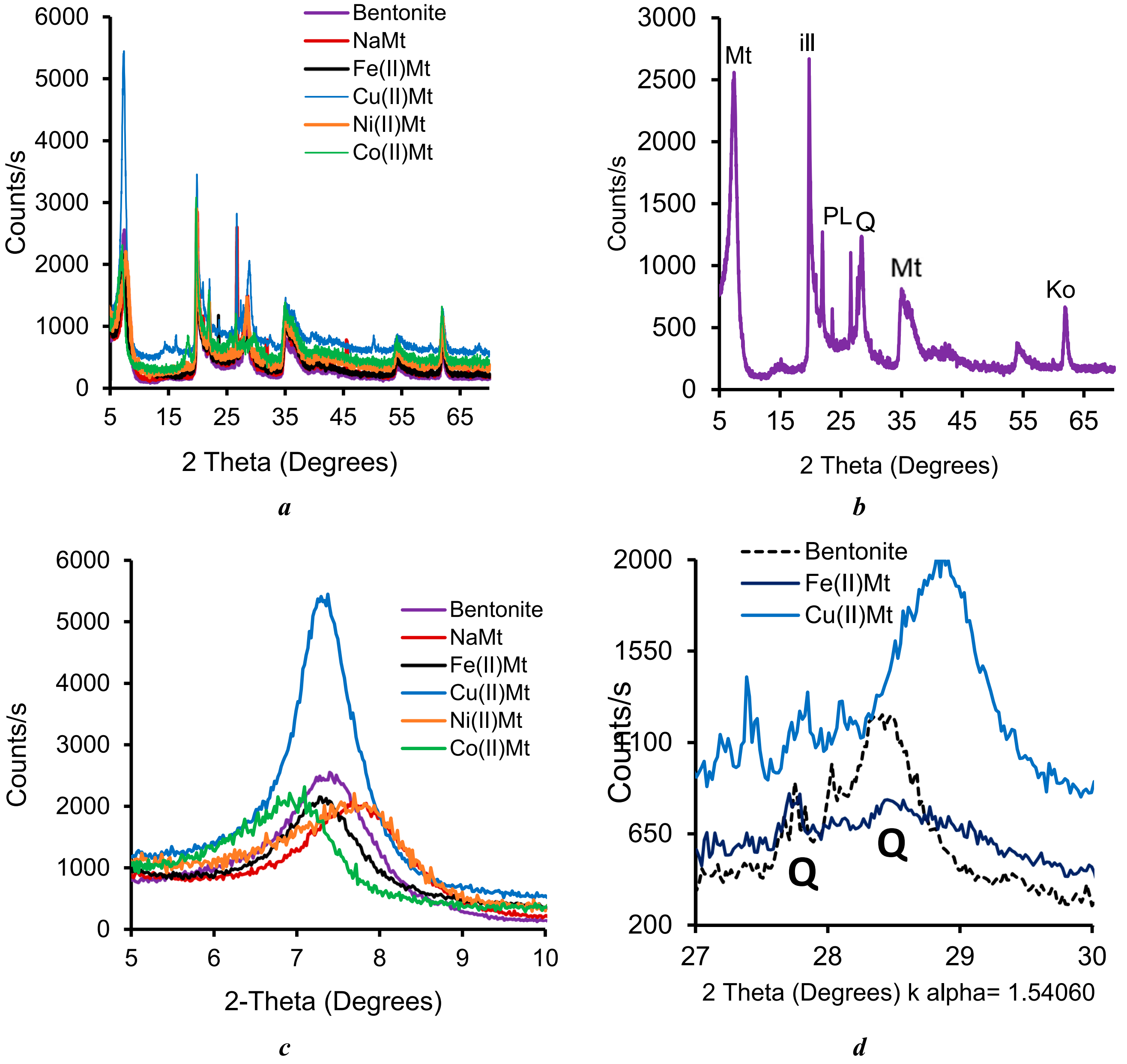

Figure S1. X-ray diffraction patterns of the clay materials investigated (a), XRD reflexion of main bentonite impurities (b) and close-ups on changes in the d_{001} basal spacing in the 2-Theta ranges 5-10 degrees (c) and 25-30 degrees for quartz removal detection after clay purification (d).

2. Concentration of dissolved ozone

An ozone generator A_2Z (A_2Z Ozone Inc., Louisville, KY, USA) with throughput **600 mg/h** was used for all ozonation experiments. The dissolved ozone concentration was then determined using the same iodometric method, applied to 20 mL samples of distilled water at different ozonation times (1, 2, 5, 10, 15, 20, 30, 40, and 60 minutes). For this purpose, ten 250 mL erlenmeyer flasks were prepared containing an acidified potassium iodide solution. The solution was prepared by dissolving 0.5 g of KI and 0.05 g of KIO_3 in 100 mL of distilled water. After complete dissolution, the volume was adjusted to 200 mL with additional distilled water. The solution was then acidified with 10 mL of 1 M HCl. Following this, 7.8 mL of 0.1 M sodium thiosulfate ($Na_2S_2O_3$) was added, resulting in a dark yellow color. Each flask was ozonated for 5 minutes at different ozone concentrations (10% to 100%). The samples were analyzed by UV–Vis spectrophotometry. The iodine (I_2) generated from the oxidation of iodide by ozone was subsequently titrated with a 0.1 M of $Na_2S_2O_3$ in the presence of 1 mL of starch as indicator until the disappearance of the blue color and the appearance of a pale-yellow endpoint. The number of moles of ozone was calculated based on the volume of thiosulfate consumed, using the following equation: $n(O_3) = [S_2O_3^{2-}] \times V(S_2O_3^{2-})/2$. The corresponding mass flow of ozone was then calculated as: $D_{mass} = n(O_3) \times M(O_3)/\ \Delta t$ where $M(O_3) = 48$ g/mol and Δt is the ozonation time. The volume of thiosulfate used is directly proportional to the amount of dissolved ozone [1]. The concentration of dissolved ozone increases almost linearly up to its maximum level of ca. 0.35-0.40 mmol/L after 10-15 min gas bubbling depending on ozone concentration in the carrier gas [2] and operating conditions [3]. This value is much lower than the reported 20.8 mmol/L in water at 0 °C [4] or 11.9 mmol/L in water at 20 °C [5-7]. The resulting O_3/NOF molar ratio of ca. 121 ensures a sufficient excess of ozone after 10 min ozonation.

3. pKa of NOF molecule and UV-Vis spectrum change during non-catalytic ozonation

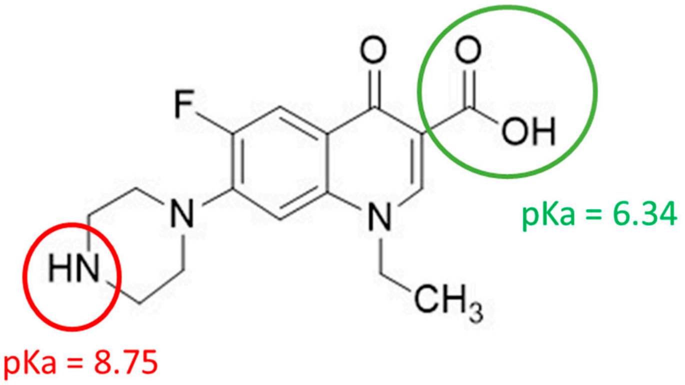

Scheme. S1. Chemical structures and related pKa constants for Norfloxacin

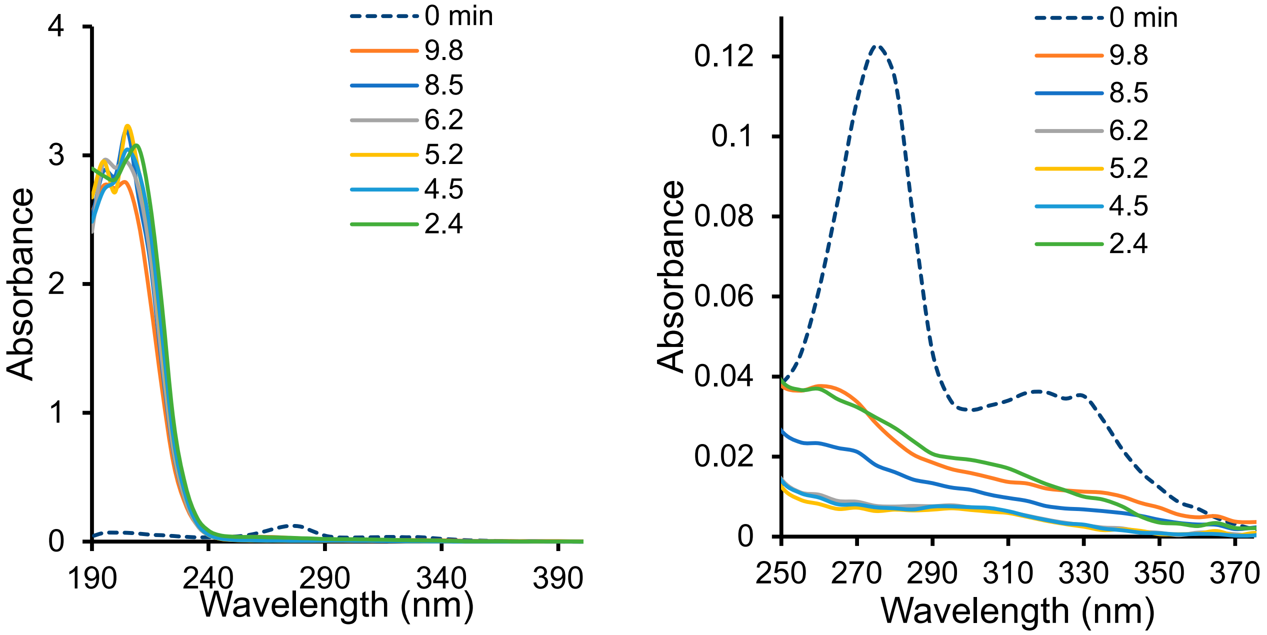

a *b*

Figure S2. pH effect on UV-Vis spectra of NOF (a) and close up on the 250-370 region (b) after 10 min of non-catalytic ozonation as compared to the non-ozonized NOF solution (Dashed line).

4. Effect of cations and exchangeable Mt samples on pH decrease during ozonation

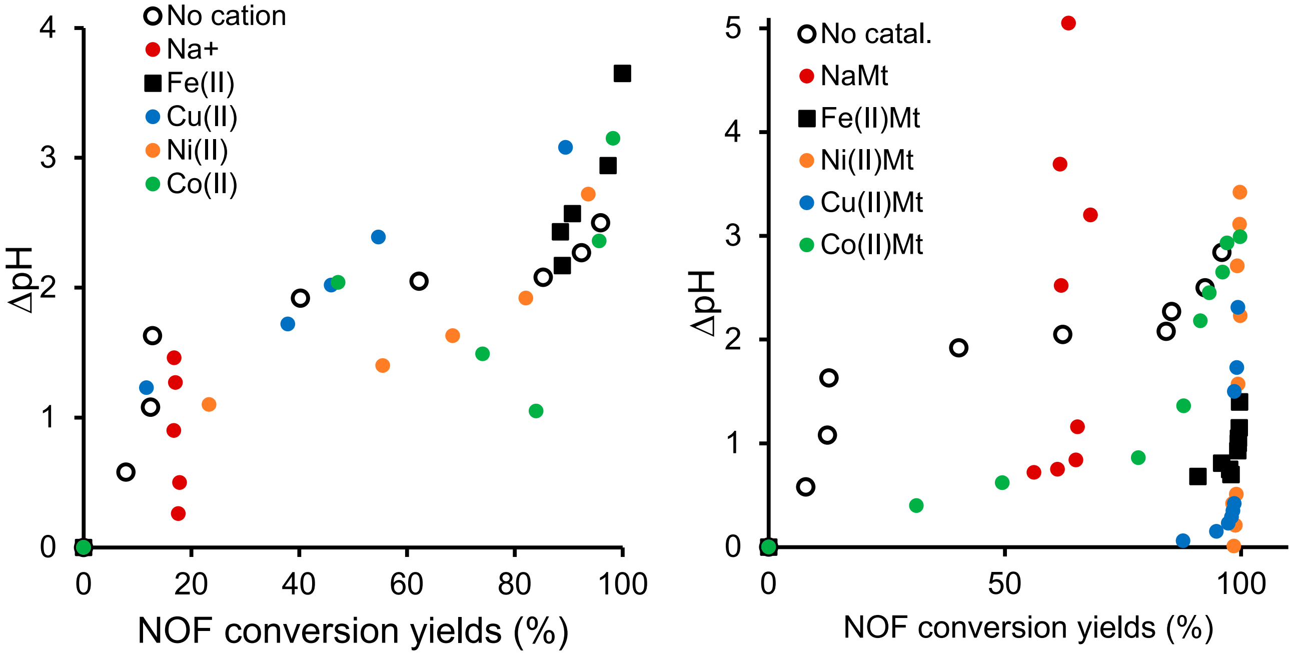

a *b*

Figure S3. pH variation (difference between initial pH and instant pH) as function of NOF conversion yield in the presence of dissolved (a) and clay-supported metal cations (b). NOF conversion was determined up to 10 min of cation-catalyzed ozonation and up to 30 min of clay-catalyzed ozonation. NOF conversion was assessed up to 30 min of non-catalytic ozonation.

5. UV-Vis spectra during Na^{+}-catalyzed NOF ozonation during ozonation

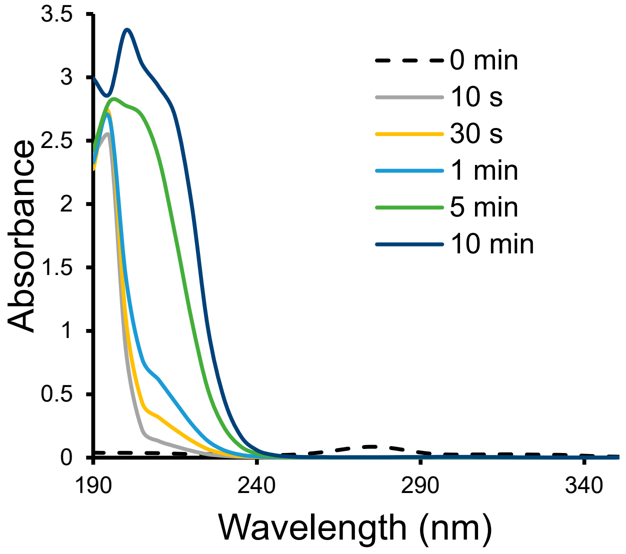

Figure S4. Evolution of UV-Vis spectra during Na^{+}-catalyzed NOF ozonation

6. pH Evolution during ozonation

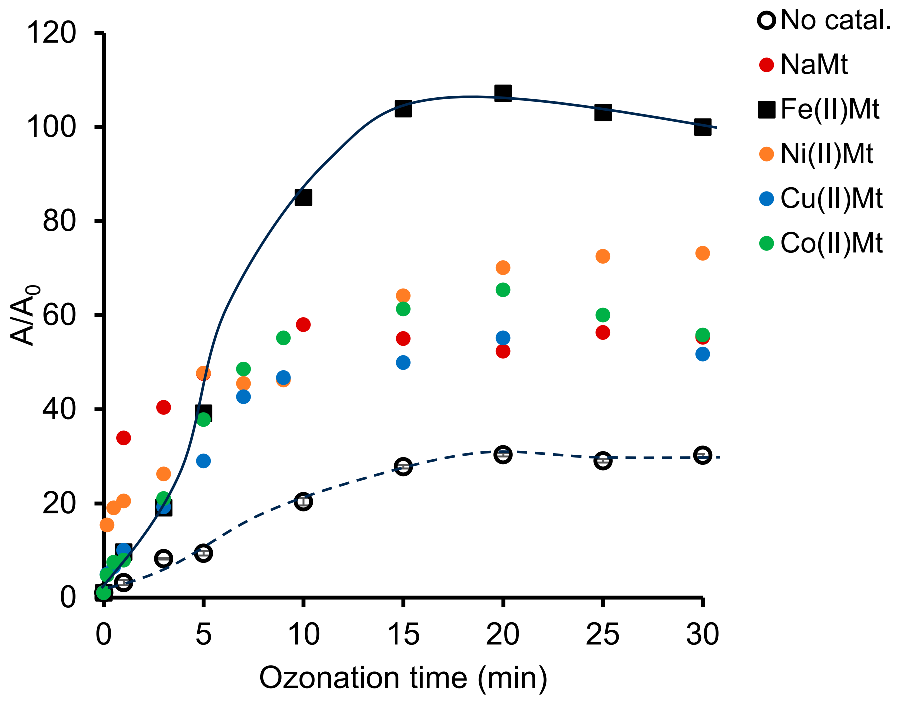

Figure S5. Evolution of the relative absorbance of the ozonized NOF solution at 200 nm (a).

7. ICP-OES measurements-operating conditions

Table S1. Instrumental parameters of ICP-OES

**Parameter**	**value**
Power	1.5 kW
Plasma argon flow rate	12 L/min
Auxiliary argon flow rate	1 L/min
Nebulizer argon flow rate	1 L/min
Wavelengths (nm)	Na (589.592), Fe (238.204), Ni (216.555), Cu (199.970) and Co (228.615).

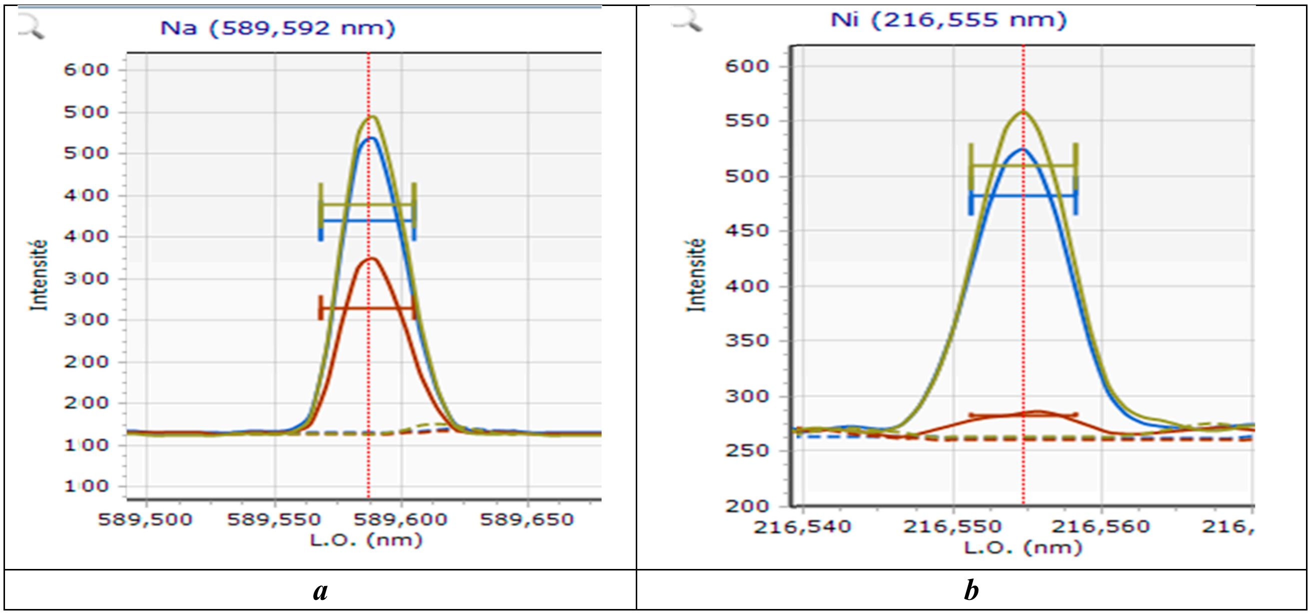

a	*b*

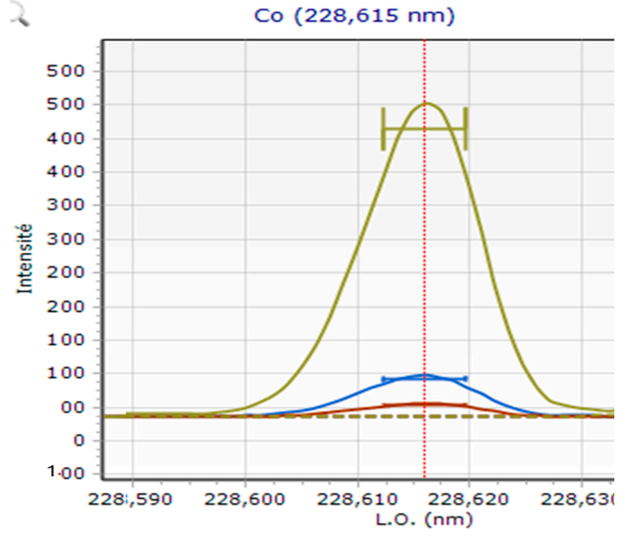

c

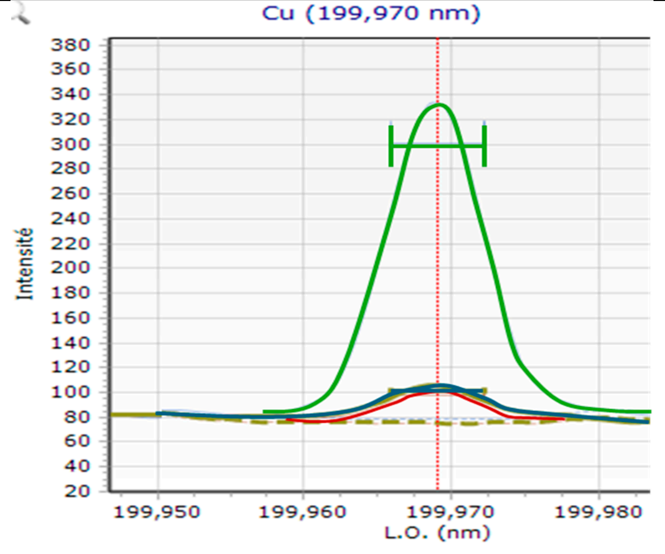

d

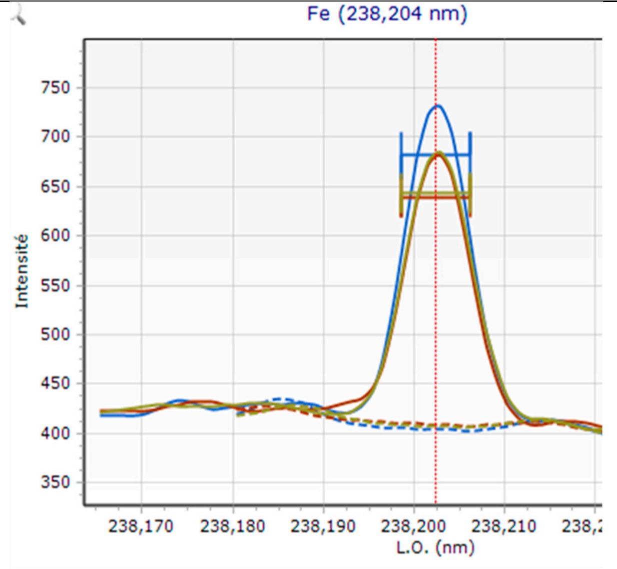

e

Figure S6. Emission spectra of different cations detected in the NOF solution by ICP-OES before and after ozonation. The blue curve corresponds to the initial time (0 min) before ozonation. The red and green curves represent the emission after 30 s and 30 min of ozonation, respectively.

8. Liquid-phase chromatography measurements-operating conditions

Table S2. Parameters of HPLC-DAD analysis

Parameter	Specification
Apparatus model	Agilent Technologies model 1290 equipment
Column specification	C18 column 4.6 ×150 mm,5 µm particle size

Mobile phases composition	A: water with 0.1% Formic Acid<br>B: methanol
Detection wavelength	275 nm
Gradient / isocratic	0-0.5 min, 10% B; 0.5-2.0 min, 10-15% B; 2.0-8.0 min, 15-35% B; 8.0-10.0 min, 35-90% B; 10.0-11.0 min, 90% B; 11.0-11.2 min, 90-10% B; 11.2-18.0 min, 10%B.
Flow rate	1 mL. min^{-1}
Temperature	20 °C
Pump pressure	18 MPa
Injected volume	20 uL

9. *HPLC-DAD measurements of residual NOF*

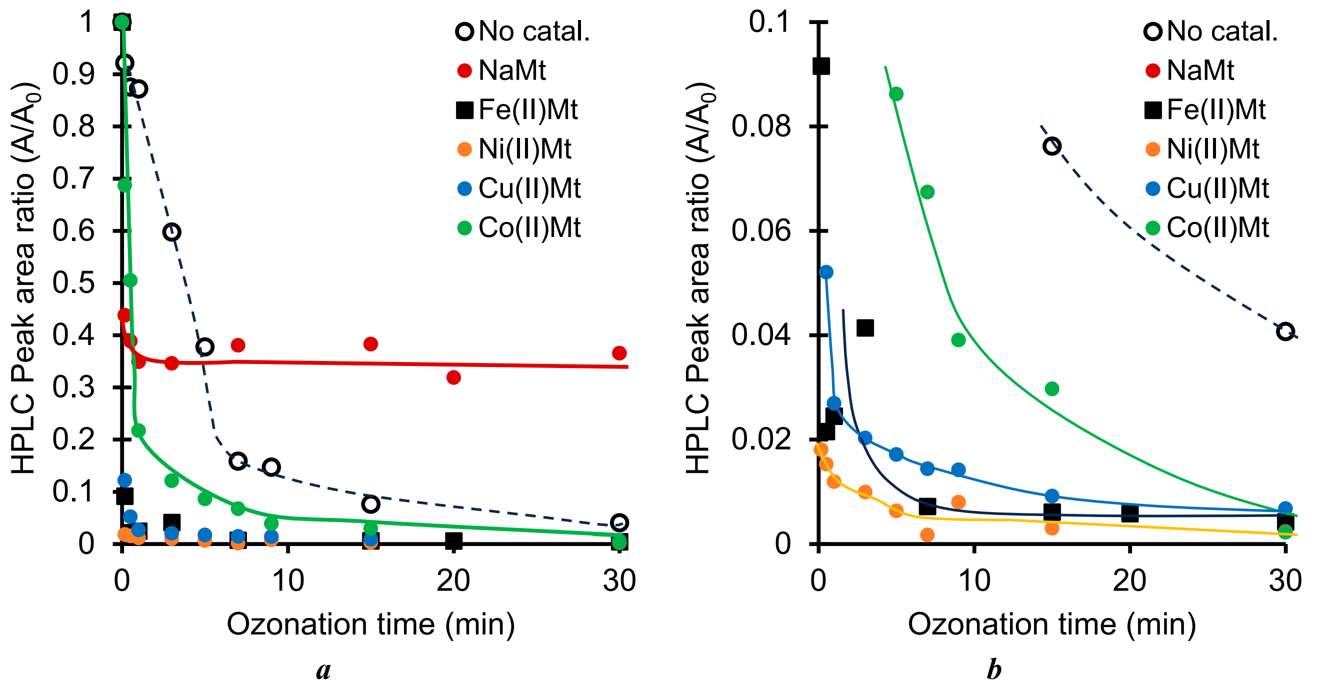

Figure S7. Evolution of the relative area of the HPLC-DAD peak of NOF during the catalytic ozonation (a) and closeup on the A/A_0 ratio range 0-0.2 (b). Ozone flow rate: 600 mg/h. Catalyst amount: 50 mg. Sample volume: 25 mL. The relative peak area (A/A_0) was calculated as the instant/initial peak area ratio (A/A_0) × 100% obtained by HPLC-DAD measurements.

10. Time evolution of pH and Zeta potential during ozonation

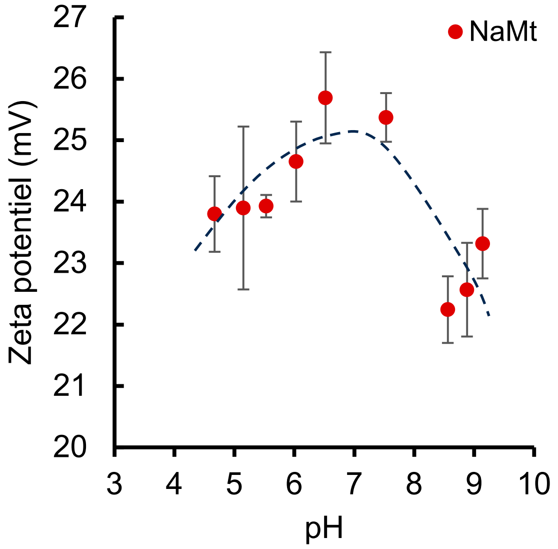

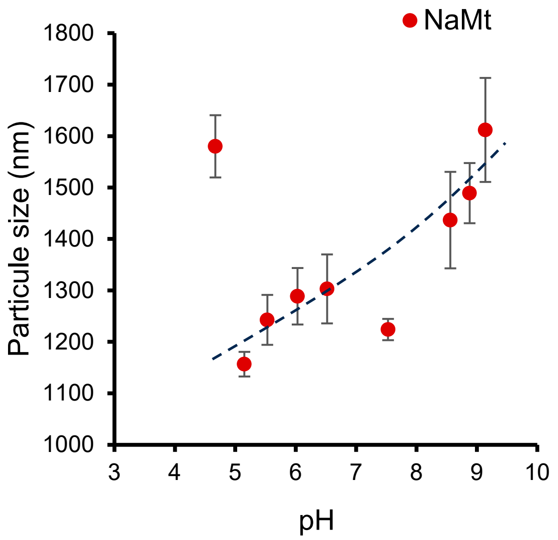

a ***b***

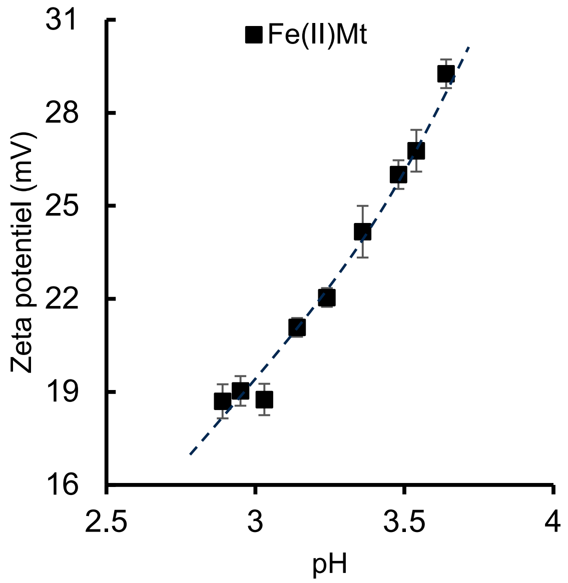

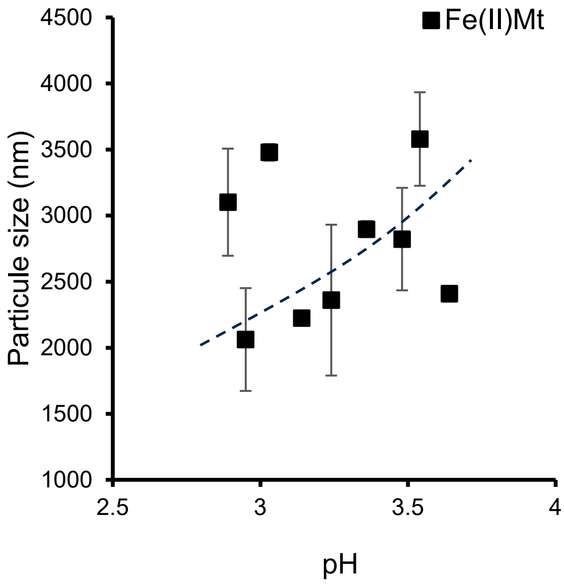

a ***b***

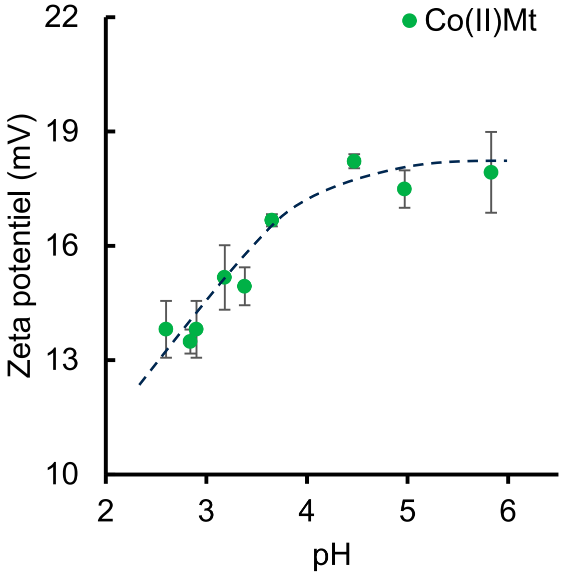
Co(II)Mt
Zeta potentiel (mV)
22
19
16
13
10
2
3
4
5
6
pH

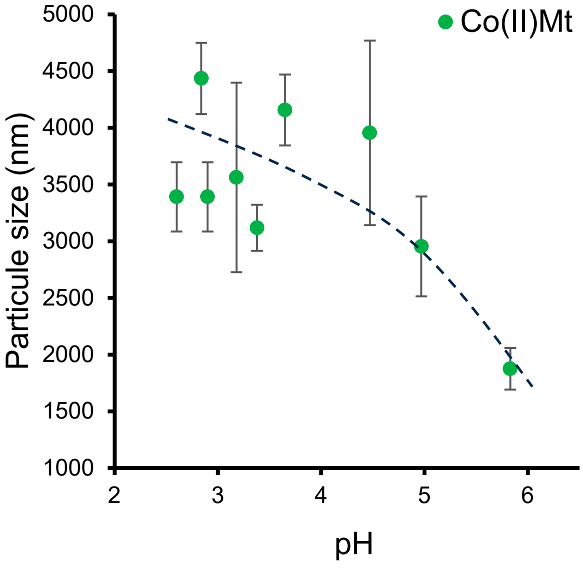
Co(II)Mt
Particule size (nm)
5000
4500
4000
3500
3000
2500
2000
1500
1000
2
3
4
5
6
pH

a *b*

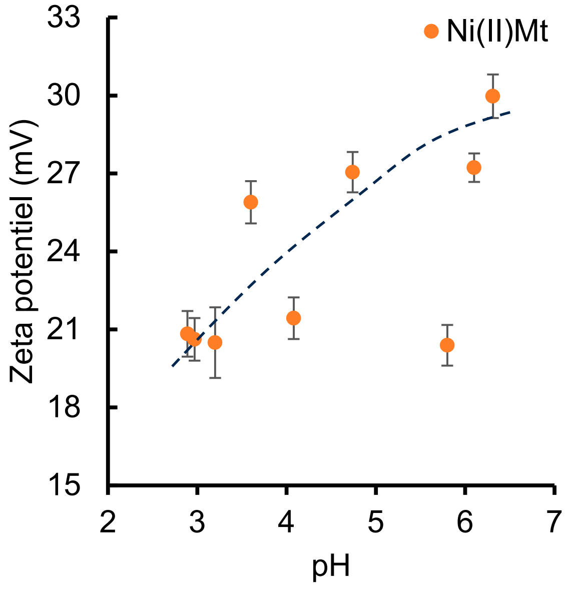
Ni(II)Mt
Zeta potentiel (mV)
33
30
27
24
21
18
15
2
3
4
5
6
7
pH

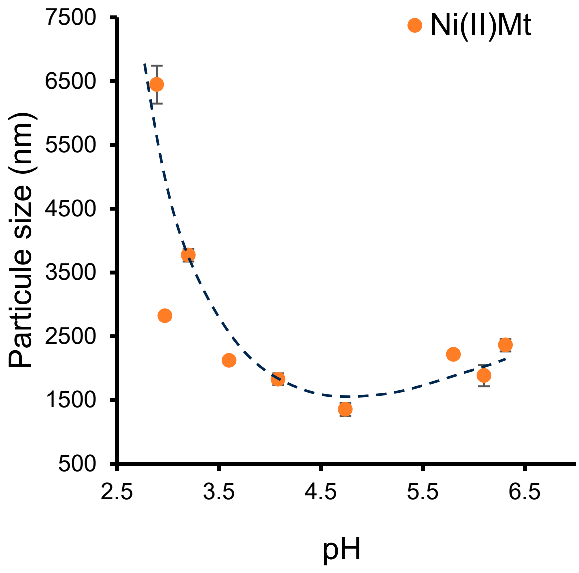
Ni(II)Mt
Particule size (nm)
7500
6500
5500
4500
3500
2500
1500
500
2.5
3.5
4.5
5.5
6.5
pH

a *a*

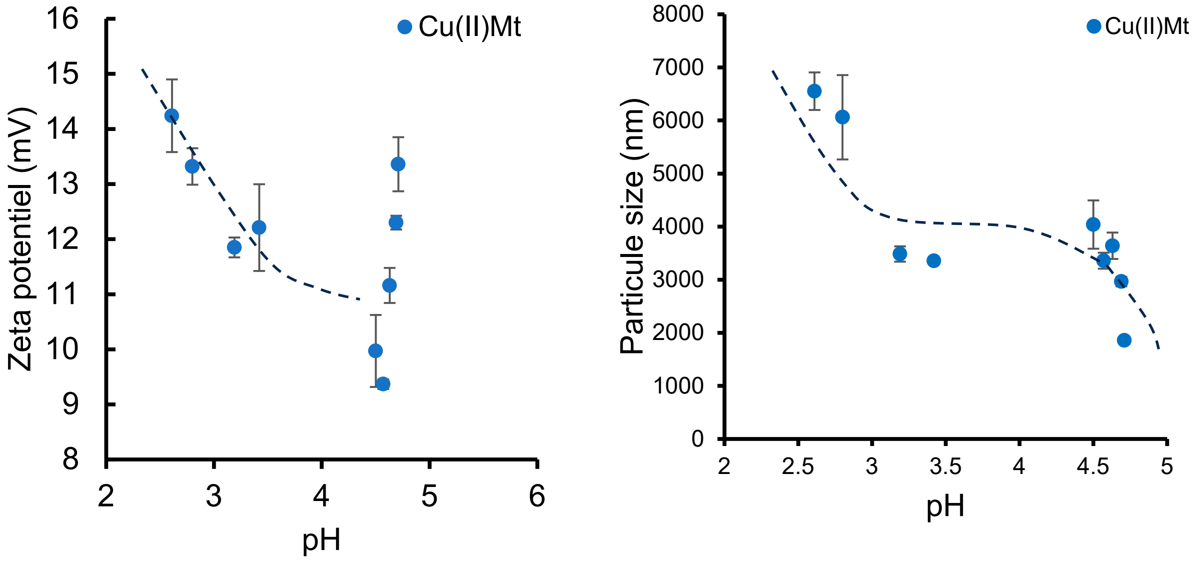

a *b*

Figure S8. Correlation between the evolution of both pH with Zeta potential (a) and with particle zize (b) during clay-catalyzed ozonation of NOF up to 30 min. Ozone flow rate: 600 mg/h. Catalysts amount: 50 mg. Sample volume: 25 mL.

11. Effect of metal cation on Chlorophyll a/b ratio

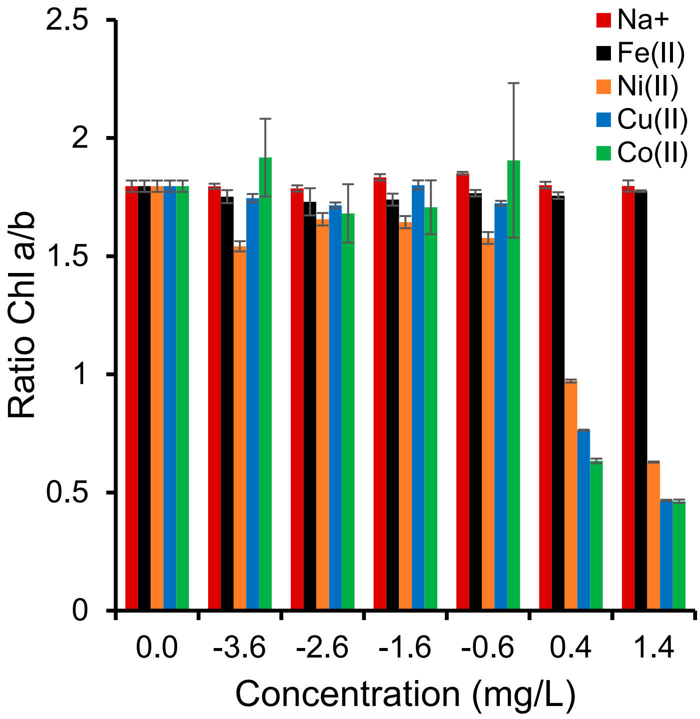

Figure S9. Effect of cations concentration on *L. minor* through changes in the chlorophyll *a/b* ratio. These data account for the mean of triplicate measurements after plant exposure for 7 days including the control sample (*Lemna minor* in SIS medium without metals).

12. The individual effects of pH and clay catalysts

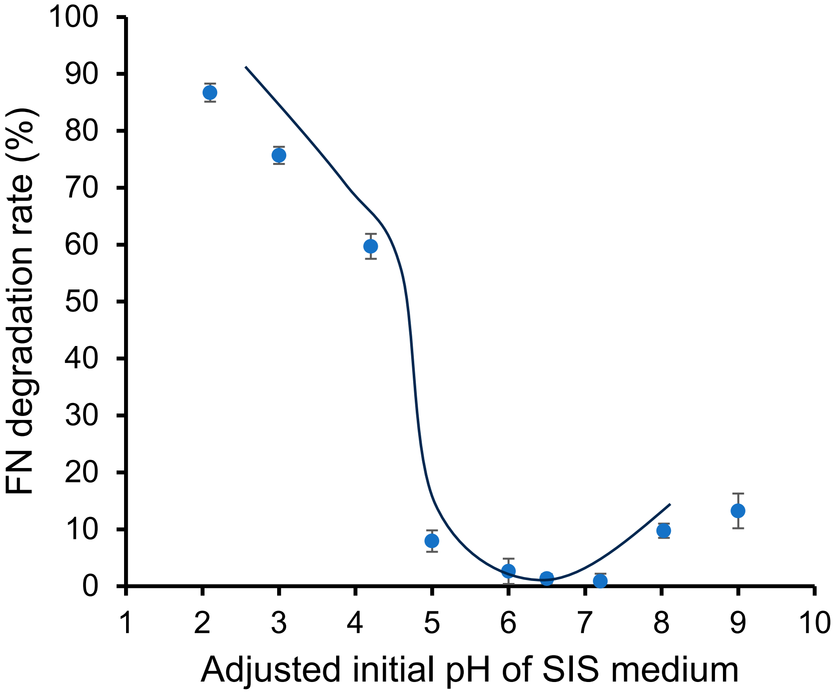

Figure S10. Effect of adjusted initial pH of SIS medium on toxicity on *L. minor* as expressed in terms of inhibition of frond number growth as reported to the blank sample (pH = 6.5). This result is obtained after 7 days of exposure in SIS medium with different pH ranging from 2 to 9. The adjusted initial pH was measured with a ± 0.1 accuracy. The results are expressed as percentages relative to the control sample.

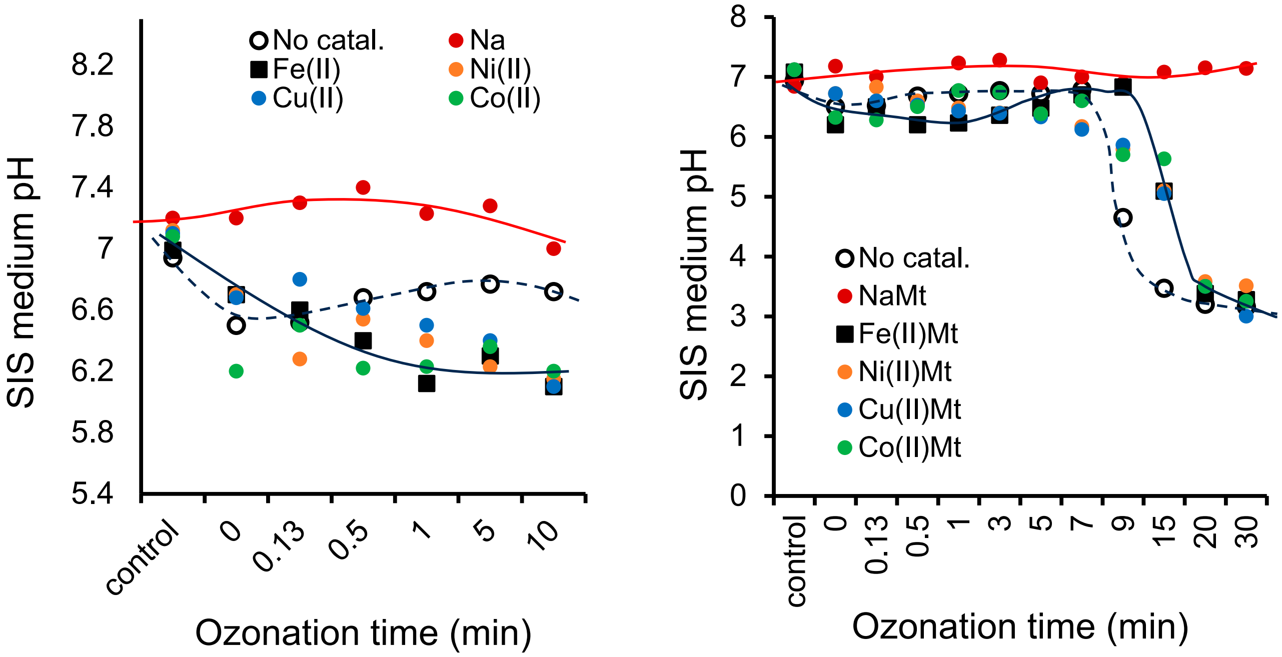

a *b*

Figure S11. pH of SIS medium after addition of reaction mixtures resulting from cation- (a) and clay-catalyzed NOF ozonation (b). The pH was measured with a ± 0.1 accuracy.

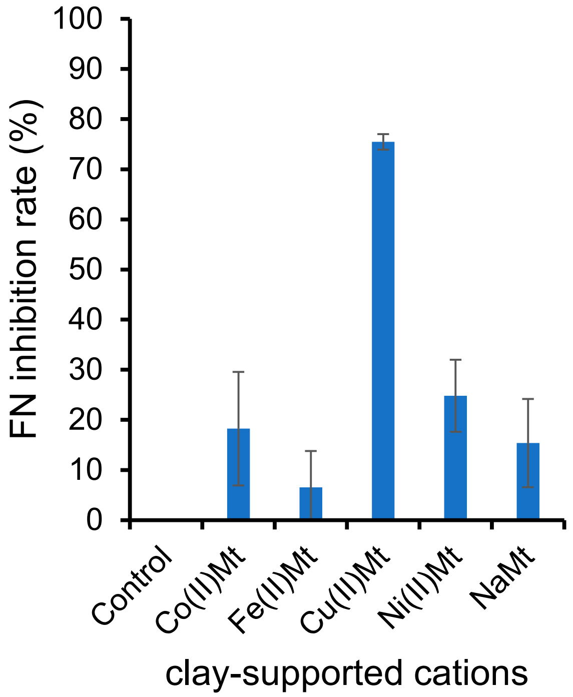

Figure S12. Effect of clay catalyst alone on toxicity on *L. minor* as expressed in terms of inhibition of frond number growth as reported to the blank sample (without clay catalyst). This result is obtained after 7 days of exposure in SIS medium. The results are expressed as percentages relative to the control sample.

13. Changes in the fast and polyphasic fluorescence kinetics of chlorophyll a of L. minor

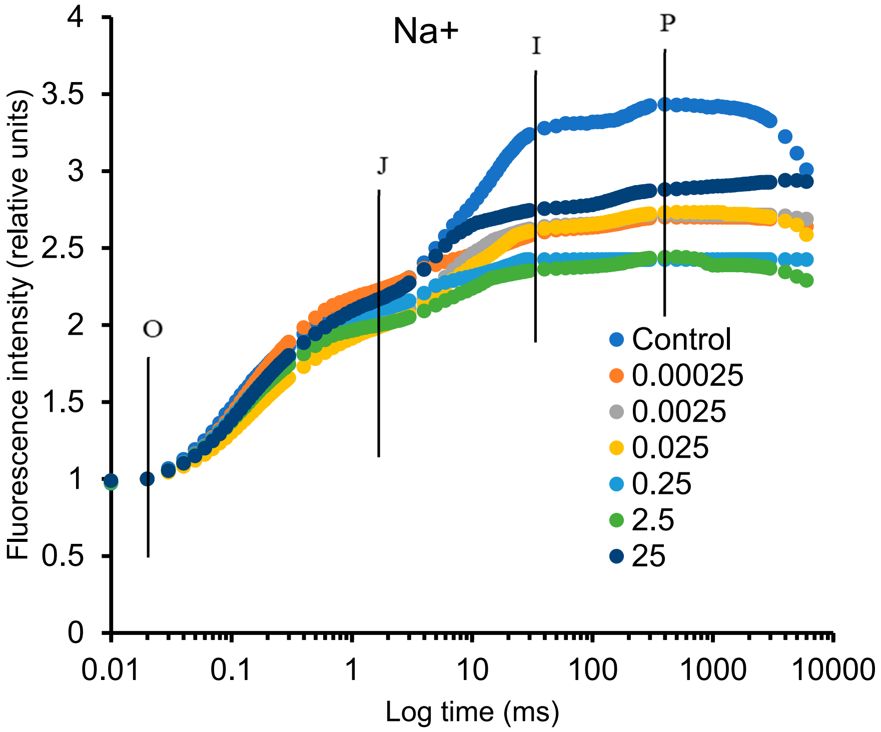

a

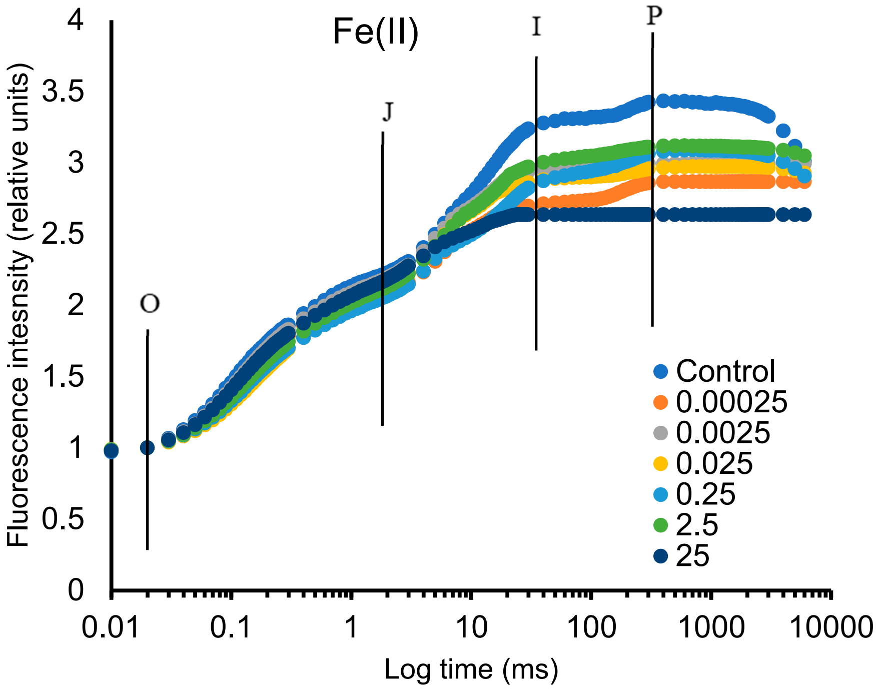
Fe(II)
Fluorescence intesnsity (relative units)
Log time (ms)
O
J
I
P
Control
0.00025
0.0025
0.025
0.25
2.5
25
0
0.5
1
1.5
2
2.5
3
3.5
4
0.01
0.1
1
10
100
1000
10000

b

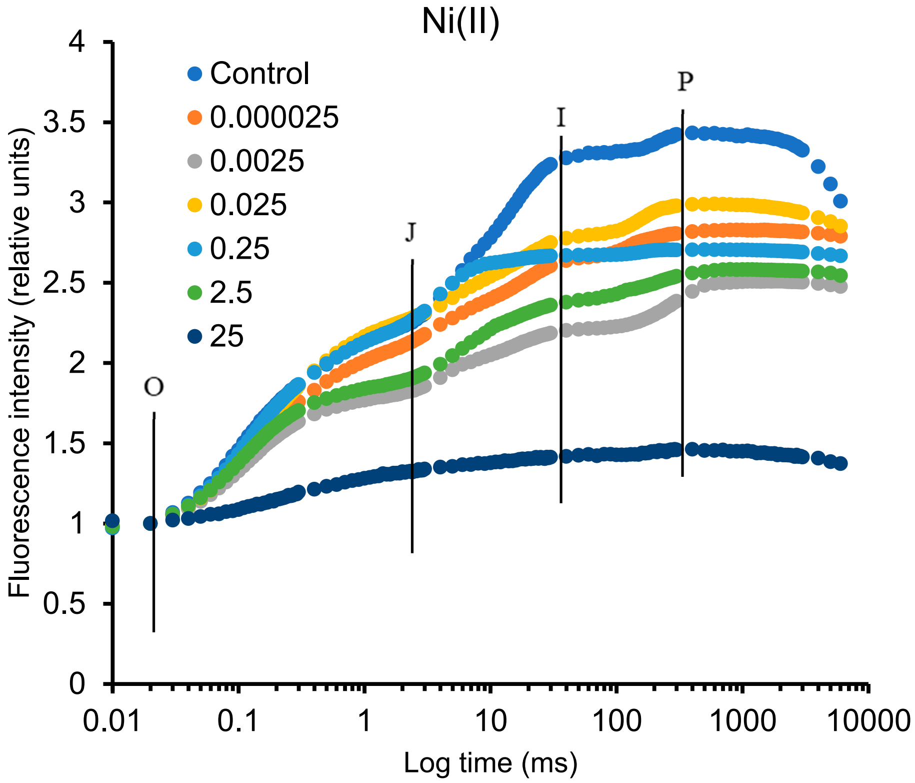
Ni(II)
Fluorescence intensity (relative units)
Log time (ms)
O
J
I
P
Control
0.000025
0.0025
0.025
0.25
2.5
25
0
0.5
1
1.5
2
2.5
3
3.5
4
0.01
0.1
1
10
100
1000
10000

c

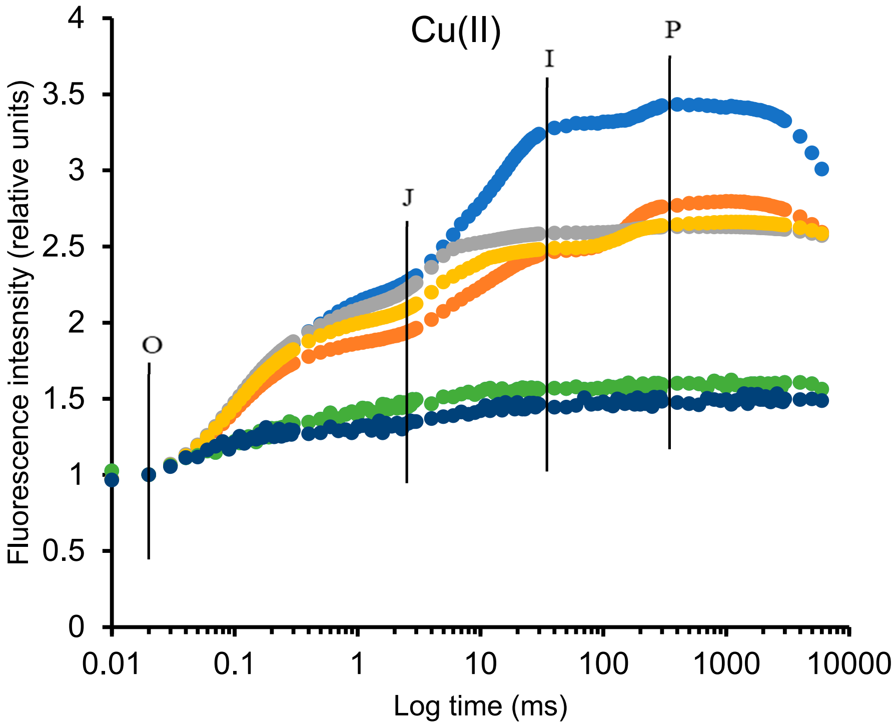

d

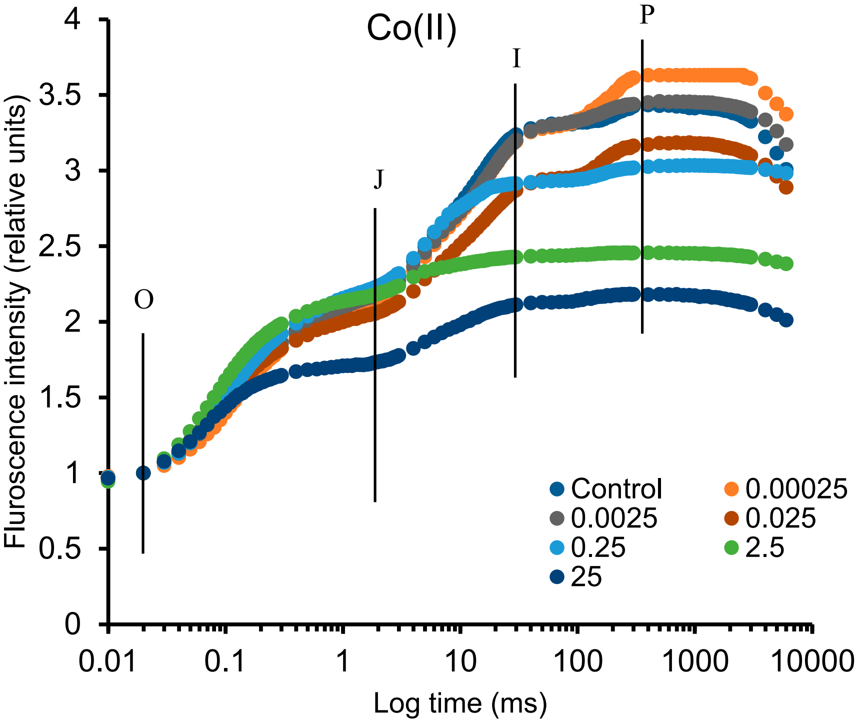

e

Figure S13. Change in the fast and polyphasic fluorescence kinetics of chlorophyll *a* of *L. minor.* Plants were exposed for 7 days to different cation concentrations (mg/L). Symbols O, J, I and P represent, respectively, the fluorescence intensities at 50 μs, 2ms, 30ms, and 200ms.

14. Changes in the variable fluorescence relative to the reduction of quinone A

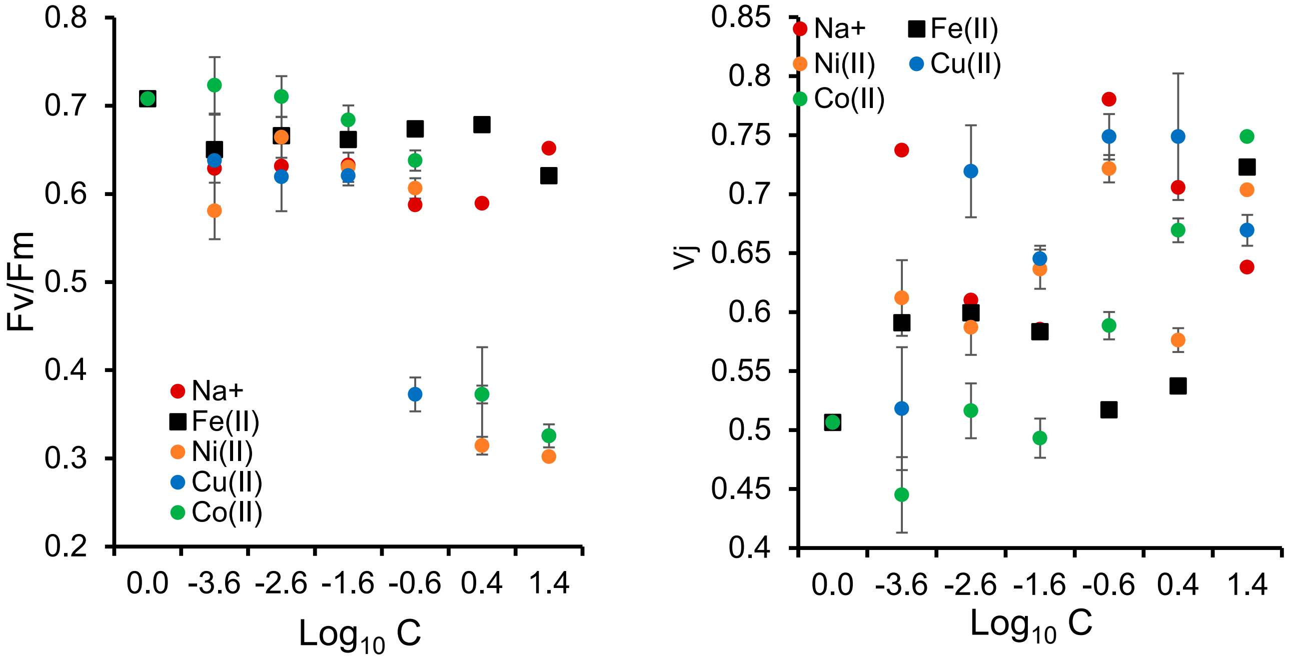

a ***b***

Figure S14. Change in the variable fluorescence relative to the reduction of quinone A Q_A (Vj). *L. minor* were exposed for 7 days to different cation concentrations (mg/L). Triplicates measurements were achieved including the metals-free control sample. The control consisted of plants grown in SIS medium, an average fresh weight of 96 mg and 66 fronds.

15. Effect of cation-catalyzed NOF ozonation on chlorophyll a/b ratio of L. minor

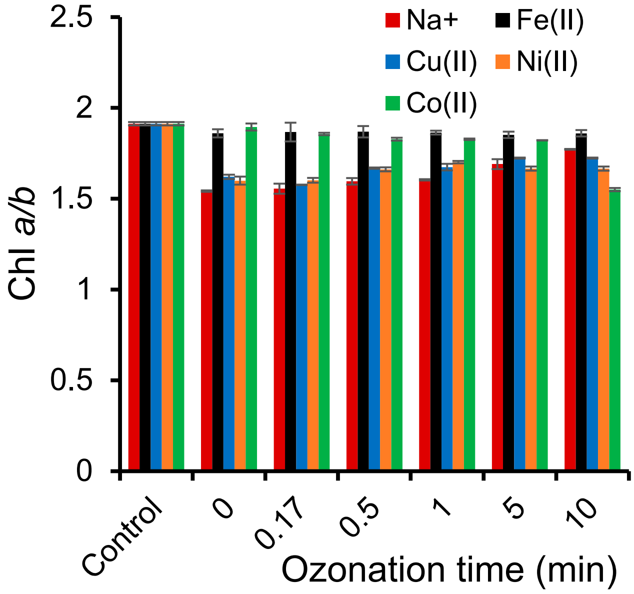

Figure S15. Effect of cation-catalyzed NOF ozonation on chlorophyll *a/b* ratio of *L. minor*. Triplicates measurements were achieved including the metals-free control sample that consisted in plants growth in SIS medium, an average fresh weight of 96 mg and 66 fronds.

16. Effect of cation-catalyzed NOF ozonation on frond number inhibition and ROS production

Table S3. ANOVA-Test of multiple comparison for concentration effect of cations alone dissolved in water on toxicity on *L. minor* as expressed in terms of frond number inhibition rate and relative ROS production

	Na^{+} cation							
	Frond number inhibition rate (%)				ROS relative production (%)			
	MeanDiff	Prob	Alpha	Sig	MeanDiff	Prob	Alpha	Sig
-3.6 0	16.66667	<0.0001	0.05	1	-56.1778	0.59172	0.05	0
-2.6 0	27.77778	<0.0001	0.05	1	-54.28077	0.62647	0.05	0
-6.2	11.11111	0.00328	0.05	1	1.89703	1	0.05	0
-1.6 0	12.12121	0.00149	0.05	1	-73.164	0.31178	0.05	0
-5.2	-4.54545	0.44853	0.05	0	-16.9862	0.99779	0.05	0
-4.2	-15.65657	1.11E-04	0.05	1	-18.88322	0.99606	0.05	0
-0.6 0	17.67677	<0.0001	0.05	1	-42.05377	0.83327	0.05	0
-4.2	1.0101	0.99916	0.05	0	14.12403	0.99921	0.05	0
-3.2	-10.10101	0.00735	0.05	1	12.227	0.99965	0.05	0
-2.2	5.55556	0.24485	0.05	0	31.11023	0.95168	0.05	0
0.4 0	19.19192	<0.0001	0.05	1	42.18887	0.83132	0.05	0
0.4 -3.6	2.52525	0.91218	0.05	0	98.36667	0.08959	0.05	0
0.4 -2.6	-8.58586	0.02492	0.05	1	96.46965	0.09918	0.05	0
0.4 -1.6	7.07071	0.08235	0.05	0	115.35287	0.03499	0.05	1
0.4 -0.6	1.51515	0.99231	0.05	0	84.24264	0.18604	0.05	0
1.4 0	24.74747	<0.0001	0.05	1	30.72668	0.95433	0.05	0
1.4 -3.6	8.08081	0.03734	0.05	1	86.90448	0.16295	0.05	0
1.4 -2.6	-3.0303	0.82196	0.05	0	85.00746	0.17914	0.05	0
1.4 -1.6	12.62626	0.00101	0.05	1	103.89068	0.06634	0.05	0
1.4 -0.6	7.07071	0.08235	0.05	0	72.78045	0.31703	0.05	0
1.4 0.4	5.55556	0.24485	0.05	0	-11.46219	0.99976	0.05	0
Fe^{2+} cation								
	Frond number inhibition rate (%)				ROS relative production (%)			
	MeanDiff	Prob	Alpha	Sig	MeanDiff	Prob	Alpha	Sig
-3.6 0	4.84092	0.91362	0.05	0	22.22785	0.99956	0.05	0
-2.6 0	18.11516	0.0128	0.05	1	-49.0475	0.96987	0.05	0
-6.2	13.27424	0.094	0.05	0	-71.27536	0.85027	0.05	0
-1.6 0	6.44788	0.74985	0.05	0	11.11619	0.99999	0.05	0
-5.2	1.60696	0.99972	0.05	0	-11.11166	0.99999	0.05	0
-4.2	-11.66728	0.17331	0.05	0	60.16369	0.92407	0.05	0
-0.6 0	4.70719	0.92324	0.05	0	-62.0553	0.91348	0.05	0
-4.2	-0.13373	1	0.05	0	-84.28315	0.73361	0.05	0
-3.2	-13.40797	0.08917	0.05	0	-13.0078	0.99998	0.05	0
-2.2	-1.74069	0.99955	0.05	0	-73.17149	0.83504	0.05	0
0.4 0	8.53208	0.47567	0.05	0	34.00555	0.99534	0.05	0
0.4 -3.6	3.69116	0.97469	0.05	0	11.7777	0.99999	0.05	0
0.4 -2.6	-9.58308	0.3514	0.05	0	83.05305	0.74568	0.05	0
0.4 -1.6	2.0842	0.99876	0.05	0	22.88936	0.99948	0.05	0
0.4 -0.6	3.82489	0.96998	0.05	0	96.06085	0.61243	0.05	0

1.4 0	5.93301	0.81083	0.05	0	-38.82035	0.99062	0.05	0
1.4 -3.6	1.0921	0.99997	0.05	0	-61.04821	0.91922	0.05	0
1.4 -2.6	-12.18215	0.14316	0.05	0	10.22715	0.99999	0.05	0
1.4 -1.6	-0.51487	1	0.05	0	-49.93654	0.96719	0.05	0
1.4 -0.6	1.22582	0.99994	0.05	0	23.23495	0.99944	0.05	0
1.4 0.4	-2.59907	0.99583	0.05	0	-72.82591	0.83787	0.05	0
Ni^{2+} cation								
	Frond number inhibition rate (%)				ROS relative production (%)			
	MeanDiff	Prob	Alpha	Sig	MeanDiff	Prob	Alpha	Sig
-3.6 0	19.69697	<0.0001	0.05	1	73.44753	0.99651	0.05	0
-2.6 0	24.74747	<0.0001	0.05	1	11.3901	1	0.05	0
-6.2	5.05051	0.12456	0.05	0	-62.05743	0.99862	0.05	0
-1.6 0	27.27273	<0.0001	0.05	1	31.2925	0.99997	0.05	0
-5.2	7.57576	0.00951	0.05	1	-42.15503	0.99985	0.05	0
-4.2	2.52525	0.77108	0.05	0	19.9024	1	0.05	0
-0.6 0	37.37374	<0.0001	0.05	1	350.42298	0.15341	0.05	0
-4.2	17.67677	<0.0001	0.05	1	276.97545	0.36233	0.05	0
-3.2	12.62626	<0.0001	0.05	1	339.03288	0.17712	0.05	0
-2.2	10.10101	7.38E-04	0.05	1	319.13048	0.22586	0.05	0
0.4 0	77.77778	<0.0001	0.05	1	774.88272	4.24E-04	0.05	1
0.4 -3.6	58.08081	<0.0001	0.05	1	701.43519	0.00113	0.05	1
0.4 -2.6	53.0303	<0.0001	0.05	1	763.49262	4.92E-04	0.05	1
0.4 -1.6	50.50505	<0.0001	0.05	1	743.59022	6.41E-04	0.05	1
0.4 -0.6	40.40404	<0.0001	0.05	1	424.45974	0.05677	0.05	0
1.4 0	86.36364	<0.0001	0.05	1	-75.84853	0.99585	0.05	0
1.4 -3.6	66.66667	<0.0001	0.05	1	-149.29606	0.89208	0.05	0
1.4 -2.6	61.61616	<0.0001	0.05	1	-87.23864	0.99128	0.05	0
1.4 -1.6	59.09091	<0.0001	0.05	1	-107.14104	0.97545	0.05	0
1.4 -0.6	48.9899	<0.0001	0.05	1	-426.27151	0.05536	0.05	0
1.4 0.4	8.58586	0.00335	0.05	1	-850.73126	1.60E-04	0.05	1
Cu^{2+} cation								
	Frond number inhibition rate (%)				ROS relative production (%)			
	MeanDiff	Prob	Alpha	Sig	MeanDiff	Prob	Alpha	Sig
-3.6 0	5.05051	0.40689	0.05	0	67.72055	0.99864	0.05	0
-2.6 0	11.61616	0.00399	0.05	1	124.5853	0.96719	0.05	0
-6.2	6.56566	0.16383	0.05	0	56.86474	0.99949	0.05	0
-1.6 0	11.61616	0.00399	0.05	1	74.26326	0.99774	0.05	0
-5.2	6.56566	0.16383	0.05	0	6.54271	1	0.05	0
-4.2	-3.02E-14	1	0.05	0	-50.32204	0.99975	0.05	0
-0.6 0	27.77778	<0.0001	0.05	1	667.01915	0.00407	0.05	1
-4.2	22.72727	<0.0001	0.05	1	599.2986	0.00982	0.05	1
-3.2	16.16162	1.62E-04	0.05	1	542.43386	0.0207	0.05	1
-2.2	16.16162	1.62E-04	0.05	1	592.75589	0.0107	0.05	1
0.4 0	80.30303	<0.0001	0.05	1	-100	0.98893	0.05	0
0.4 -3.6	75.25253	<0.0001	0.05	1	-167.72055	0.88032	0.05	0
0.4 -2.6	68.68687	<0.0001	0.05	1	-224.5853	0.67548	0.05	0
0.4 -1.6	68.68687	<0.0001	0.05	1	-174.26326	0.86124	0.05	0

0.4 -0.6	52.52525	<0.0001	0.05	1	-767.01915	0.00114	0.05	1
1.4 0	86.86869	<0.0001	0.05	1	-100	0.98893	0.05	0
1.4 -3.6	81.81818	<0.0001	0.05	1	-167.72055	0.88032	0.05	0
1.4 -2.6	75.25253	<0.0001	0.05	1	-224.5853	0.67548	0.05	0
1.4 -1.6	75.25253	<0.0001	0.05	1	-174.26326	0.86124	0.05	0
1.4 -0.6	59.09091	<0.0001	0.05	1	-767.01915	0.00114	0.05	1
1.4 0.4	6.56566	0.16383	0.05	0	0	1	0.05	0
Co^{2+} cation								
	Frond number inhibition rate (%)				ROS relative production (%)			
	MeanDiff	Prob	Alpha	Sig	MeanDiff	Prob	Alpha	Sig
-3.6 0	-3.0303	0.96629	0.05	0	-206.76957	0.73486	0.05	0
-2.6 0	2.52525	0.9862	0.05	0	-299.11749	0.36039	0.05	0
-6.2	5.55556	0.65416	0.05	0	-92.34792	0.99207	0.05	0
-1.6 0	9.59596	0.13127	0.05	0	-499.70275	0.03271	0.05	1
-5.2	12.62626	0.02702	0.05	1	-292.93318	0.38236	0.05	0
-4.2	7.07071	0.4007	0.05	0	-200.58525	0.75934	0.05	0
-0.6 0	21.21212	3.01E-04	0.05	1	4.98032	1	0.05	0
-4.2	24.24242	<0.0001	0.05	1	211.74989	0.71466	0.05	0
-3.2	18.68687	0.00106	0.05	1	304.09781	0.34327	0.05	0
-2.2	11.61616	0.04643	0.05	1	504.68307	0.03063	0.05	1
0.4 0	57.57576	<0.0001	0.05	1	517.78574	0.02575	0.05	1
0.4 -3.6	60.60606	<0.0001	0.05	1	724.5553	0.00169	0.05	1
0.4 -2.6	55.05051	<0.0001	0.05	1	816.90323	5.31E-04	0.05	1
0.4 -1.6	47.9798	<0.0001	0.05	1	1017.48848	<0.0001	0.05	1
0.4 -0.6	36.36364	<0.0001	0.05	1	512.80541	0.02751	0.05	1
1.4 0	83.33333	<0.0001	0.05	1	365.99016	0.17603	0.05	0
1.4 -3.6	86.36364	<0.0001	0.05	1	572.75972	0.01239	0.05	1
1.4 -2.6	80.80808	<0.0001	0.05	1	665.10765	0.00365	0.05	1
1.4 -1.6	73.73737	<0.0001	0.05	1	865.6929	2.94E-04	0.05	1
1.4 -0.6	62.12121	<0.0001	0.05	1	361.00983	0.18644	0.05	0
1.4 0.4	25.75758	<0.0001	0.05	1	-151.79558	0.91507	0.05	0

Table S4. ANOVA-Test of multiple comparison with control samples for Toxicity of ozonized NOF solution in presence of cations on frond number inhibition rate and ROS production.

Na^{+} cation								
	Frond number inhibition rate (%)				ROS relative production (%)			
	MeanDiff	Prob	Alpha	Sig	MeanDiff	Prob	Alpha	Sig
0 control	46.52958	<0.0001	0.05	1	142.1334	0.01617	0.05	1
0.17 control	57.17653	<0.0001	0.05	1	-52.55932	0.74427	0.05	0
0.17 0	10.64695	0.06663	0.05	0	-194.69272	0.00112	0.05	1
0.5 control	58.11688	<0.0001	0.05	1	234.59696	1.69E-04	0.05	1
0.5 0	11.5873	0.04	0.05	1	92.46355	0.18945	0.05	0
0.5 0.17	0.94036	0.99993	0.05	0	287.15628	<0.0001	0.05	1
1 control	56.59452	<0.0001	0.05	1	-43.41082	0.86968	0.05	0
1 0	10.06494	0.09071	0.05	0	-185.54422	0.00175	0.05	1

1 0.17	-0.58201	1	0.05	0	9.1485	0.99996	0.05	0
1 0.5	-1.52237	0.99896	0.05	0	-278.00777	<0.0001	0.05	1
5 control	25.73834	<0.0001	0.05	1	-61.11065	0.60536	0.05	0
5 0	-20.79125	2.82E-04	0.05	1	-203.24406	7.35E-04	0.05	1
5 0.17	-31.43819	<0.0001	0.05	1	-8.55133	0.99998	0.05	0
5 0.5	-32.37855	<0.0001	0.05	1	-295.70761	<0.0001	0.05	1
5 1	-30.85618	<0.0001	0.05	1	-17.69984	0.99838	0.05	0
10 control	56.06542	<0.0001	0.05	1	-57.86752	0.65912	0.05	0
10 0	9.53583	0.11928	0.05	0	-200.00093	8.61E-04	0.05	1
10 0.17	-1.11111	0.99983	0.05	0	-5.3082	1	0.05	0
10 0.5	-2.05147	0.99464	0.05	0	-292.46448	<0.0001	0.05	1
10 1	-0.5291	1	0.05	0	-14.45671	0.99948	0.05	0
10 5	30.32708	<0.0001	0.05	1	3.24313	1	0.05	0
Fe^{2+} cation								
	Frond number inhibition rate (%)				ROS relative production (%)			
	MeanDiff	Prob	Alpha	Sig	MeanDiff	Prob	Alpha	Sig
0 control	13.52573	0.1071	0.05	0	-49.73304	0.80846	0.05	0
0.17 control	16.75565	0.03062	0.05	1	33.63441	0.96174	0.05	0
0.17 0	3.22992	0.9896	0.05	0	83.36745	0.31008	0.05	0
0.5 control	12.46513	0.15756	0.05	0	-59.76746	0.65818	0.05	0
0.5 0	-1.06061	0.99998	0.05	0	-10.03442	0.99995	0.05	0
0.5 0.17	-4.29052	0.95818	0.05	0	-93.40187	0.20642	0.05	0
1 control	16.67869	0.03157	0.05	1	-4.36682	1	0.05	0
1 0	3.15296	0.99082	0.05	0	45.36623	0.86315	0.05	0
1 0.17	-0.07696	1	0.05	0	-38.00123	0.9337	0.05	0
1 0.5	4.21356	0.96154	0.05	0	55.40064	0.72659	0.05	0
5 control	2.09235	0.99901	0.05	0	-26.3936	0.98834	0.05	0
5 0	-11.43338	0.22488	0.05	0	23.33944	0.99386	0.05	0
5 0.17	-14.6633	0.06961	0.05	0	-60.02801	0.65402	0.05	0
5 0.5	-10.37278	0.31577	0.05	0	33.37386	0.96311	0.05	0
5 1	-14.58634	0.0717	0.05	0	-22.02678	0.99549	0.05	0
10 control	14.7595	0.06707	0.05	0	-59.61038	0.66069	0.05	0
10 0	1.23377	0.99995	0.05	0	-9.87734	0.99995	0.05	0
10 0.17	-1.99615	0.99924	0.05	0	-93.24479	0.2078	0.05	0
10 0.5	2.29437	0.99835	0.05	0	0.15708	1	0.05	0
10 1	-1.91919	0.99939	0.05	0	-55.24357	0.72898	0.05	0
10 5	12.66715	0.1466	0.05	0	-33.21678	0.96391	0.05	0
Ni^{2+} cation								
	Frond number inhibition rate (%)				ROS relative production (%)			
	MeanDiff	Prob	Alpha	Sig	MeanDiff	Prob	Alpha	Sig
0 control	30.53391	<0.0001	0.05	1	718.51112	<0.0001	0.05	1
0.17 control	27.91727	2.30E-04	0.05	1	83.36841	0.92862	0.05	0
0.17 0	-2.61664	0.99548	0.05	0	-635.14272	<0.0001	0.05	1
0.5 control	25.3824	6.09E-04	0.05	1	252.76956	0.06954	0.05	0
0.5 0	-5.15152	0.88497	0.05	0	-465.74156	5.50E-04	0.05	1
0.5 0.17	-2.53487	0.99619	0.05	0	169.40116	0.37116	0.05	0
1 control	18.34536	0.01091	0.05	1	33.22784	0.99938	0.05	0

1 0	-12.18855	0.13751	0.05	0	-685.28328	<0.0001	0.05	1
1 0.17	-9.57191	0.34412	0.05	0	-50.14056	0.99399	0.05	0
1 0.5	-7.03704	0.66622	0.05	0	-219.54172	0.14292	0.05	0
5 control	33.88648	<0.0001	0.05	1	489.28062	3.34E-04	0.05	1
5 0	3.35257	0.9836	0.05	0	-229.23051	0.11644	0.05	0
5 0.17	5.96922	0.80117	0.05	0	405.91221	0.00205	0.05	1
5 0.5	8.50409	0.47033	0.05	0	236.51105	0.09952	0.05	0
5 1	15.54113	0.03558	0.05	1	456.05277	6.78E-04	0.05	1
10 control	84.10293	<0.0001	0.05	1	-59.61038	0.98526	0.05	0
10 0	53.56902	<0.0001	0.05	1	-778.1215	<0.0001	0.05	1
10 0.17	56.18567	<0.0001	0.05	1	-142.97879	0.55338	0.05	0
10 0.5	58.72054	<0.0001	0.05	1	-312.37994	0.01771	0.05	1
10 1	65.75758	<0.0001	0.05	1	-92.83822	0.8885	0.05	0
10 5	50.21645	<0.0001	0.05	1	-548.891	<0.0001	0.05	1
Cu^{2+} cation								
	Frond number inhibition rate (%)				ROS relative production (%)			
	MeanDiff	Prob	Alpha	Sig	MeanDiff	Prob	Alpha	Sig
0 control	31.64502	0.00108	0.05	1	411.8555	0.00503	0.05	1
0.17 control	24.12939	0.01132	0.05	1	810.90537	<0.0001	0.05	1
0.17 0	-7.51563	0.83255	0.05	0	399.04987	0.00655	0.05	1
0.5 control	22.60462	0.01843	0.05	1	469.434	0.00157	0.05	1
0.5 0	-9.0404	0.69237	0.05	0	57.57849	0.99325	0.05	0
0.5 0.17	-1.52477	0.99996	0.05	0	-341.47137	0.02155	0.05	1
1 control	16.12314	0.13691	0.05	0	17.34731	0.99999	0.05	0
1 0	-15.52189	0.16253	0.05	0	-394.50819	0.00719	0.05	1
1 0.17	-8.00625	0.79082	0.05	0	-793.55806	<0.0001	0.05	1
1 0.5	-6.48148	0.90567	0.05	0	-452.08668	0.00222	0.05	1
5 control	21.37326	0.0273	0.05	1	-20.8978	0.99998	0.05	0
5 0	-10.27177	0.56676	0.05	0	-432.7533	0.00328	0.05	1
5 0.17	-2.75613	0.9987	0.05	0	-831.80317	<0.0001	0.05	1
5 0.5	-1.23136	0.99999	0.05	0	-490.33179	0.00104	0.05	1
5 1	5.25012	0.96268	0.05	0	-38.24511	0.99929	0.05	0
10 control	84.10293	<0.0001	0.05	1	-59.61038	0.9919	0.05	0
10 0	52.45791	<0.0001	0.05	1	-471.46589	0.00151	0.05	1
10 0.17	59.97354	<0.0001	0.05	1	-870.51575	<0.0001	0.05	1
10 0.5	61.49832	<0.0001	0.05	1	-529.04438	4.92E-04	0.05	1
10 1	67.9798	<0.0001	0.05	1	-76.95769	0.97081	0.05	0
10 5	62.72968	<0.0001	0.05	1	-38.71258	0.99924	0.05	0
Co^{2+} cation								
	Frond number inhibition rate (%)				ROS relative production (%)			
	MeanDiff	Prob	Alpha	Sig	MeanDiff	Prob	Alpha	Sig
0 control	27.50601	0.00192	0.05	1	55.29931	0.80159	0.05	0
0.17 control	25.20683	0.00417	0.05	1	119.6975	0.10387	0.05	0
0.17 0	-2.29918	0.99927	0.05	0	64.39819	0.67824	0.05	0
0.5 control	23.18903	0.00834	0.05	1	3.80722	1	0.05	0
0.5 0	-4.31698	0.97871	0.05	0	-51.49209	0.84639	0.05	0

0.5 0.17	-2.0178	0.99965	0.05	0	-115.89028	0.12192	0.05	0
1 control	21.22174	0.01647	0.05	1	106.63631	0.17771	0.05	0
1 0	-6.28427	0.88564	0.05	0	51.33699	0.8481	0.05	0
1 0.17	-3.98509	0.98571	0.05	0	-13.0612	0.99986	0.05	0
1 0.5	-1.96729	0.9997	0.05	0	102.82909	0.20625	0.05	0
5 control	5.17316	0.94998	0.05	0	-39.77824	0.94689	0.05	0
5 0	-22.33285	0.01121	0.05	1	-95.07755	0.27563	0.05	0
5 0.17	-20.03367	0.02483	0.05	1	-159.47574	0.01767	0.05	1
5 0.5	-18.01587	0.04949	0.05	1	-43.58546	0.92089	0.05	0
5 1	-16.04858	0.09509	0.05	0	-146.41454	0.03199	0.05	1
10 control	68.86243	<0.0001	0.05	1	228.12899	8.25E-04	0.05	1
10 0	41.35642	<0.0001	0.05	1	172.82968	0.00961	0.05	1
10 0.17	43.6556	<0.0001	0.05	1	108.43149	0.16544	0.05	0
10 0.5	45.6734	<0.0001	0.05	1	224.32177	9.72E-04	0.05	1
10 1	47.64069	<0.0001	0.05	1	121.49269	0.09623	0.05	0
10 5	63.68927	<0.0001	0.05	1	267.90723	1.60E-04	0.05	1

1. Chasanah, U., et al. *Evaluation of titration method on determination of ozone concentration produced by dielectric barrier discharge plasma (DBDP) technology*. in *Journal of physics: conference series*. 2019. IOP Publishing.
2. Benghaffour, A., et al., *Insight in natural media remediation through ecotoxicity correlation to clay catalyst selectivity in organic molecule ozonation.* Dalton Transactions, 2022. **51**(11): p. 4366-4376.
3. Zekkari, M., et al., *Silica-catalyzed ozonation of 17α-ethinyl-estradiol in aqueous media-to better understand the role of silica in soils.* Chemosphere, 2022. **298**: p. 134312.
4. Battino, R., T.R. Rettich, and T. Tominaga, *The solubility of oxygen and ozone in liquids.* Journal of physical and chemical reference data, 1983. **12**(2): p. 163-178.
5. Shahidi, D., R. Roy, and A. Azzouz, *Advances in catalytic oxidation of organic pollutants–prospects for thorough mineralization by natural clay catalysts.* Applied Catalysis B: Environmental, 2015. **174**: p. 277-292.
6. Egorova, G., et al., *Ozone solubility in water.* Moscow University Chemistry Bulletin, 2015. **70**: p. 207-210.
7. Roth, J.A. and D.E. Sullivan, *Solubility of ozone in water.* Industrial & Engineering Chemistry Fundamentals, 1981. **20**(2): p. 137-140.
